# Study on the fracture behavior and critical slowing down characteristics of saturated sandstone based on acoustic emission and resistivity

**DOI:** 10.1038/s41598-025-96072-w

**Published:** 2025-04-09

**Authors:** Yantao Zheng, Changwu Liu, Kunpeng Lu, Hexing Zhang, Bingxi Jian, Wuzhou Zhang

**Affiliations:** 1 College of Water Resource and Hydropower, Sichuan University, Chengdu, 610065 China; 2 Guizhou Chengqian Mining Development Co., Ltd., Guiyang, 550000 China; 3 Key Laboratory of Karst Georesources and Environment (Guizhou University), College of Resources and Environmental Engineering, Ministry of Education, Guiyang, 550025 China; 4 School of Civil Engineering, Chongqing University, Chongqing, 400045 China

**Keywords:** Saturated sandstone, Acoustic emission, Resistivity, Fracture behavior, Critical slowing down theory, Geology, Civil engineering

## Abstract

Water significantly affects the fracture behavior and acoustic emission (AE) characteristics of sandstone, which is crucial for assessing the stability of underground engineering. This study investigates the fracture behavior of four types of saturated sandstone (green, white, brown, and red) using AE and resistivity monitoring techniques during uniaxial compression tests. AE energy, resistivity, and RA–AF parameters were analyzed to comprehensively assess the fracture behavior. Additionally, precursor information of sandstone failure was examined using the critical slowing down theory. The results revealed a decrease in uniaxial compressive strength and elastic modulus in saturated sandstone, with water softening effects ranked as brown, red, white, and green sandstone. Resistivity variation was highly sensitive to fracture development and demonstrated strong complementarity with AE signals. Crack classification based on the RA–AF parameter aligned with macroscopic failure patterns. Water presence accelerated the initiation of tensile cracks, with growth rates of 13.66%, 13.69%, 14.86%, and 17.53%, correlating with porosity and pore water pressure. The sharp increase in the autocorrelation coefficient and variance of AE parameters (amplitude, RA value, rise time, AE energy) before critical failure indicated a critical slowing down phenomenon, serving as a potential precursor to sandstone failure. Moreover, the sensitivity and reliability of critical slowing down theory in early warning applications were affected by water and porosity, which should be considered in practice.

## Introduction

As urban underground space development, nuclear waste disposal, deep mineral resource exploration, and underground gas storage projects delve deeper into the subsurface, the challenges and scale facing underground rock and soil engineering continue to grow, with safety concerns being particularly prominent. Particularly as depth increases, rock formations often find themselves in hydrostatic conditions. The infiltration of water not only leads to the dissolution and reorganization of rock mineral structures but also triggers changes in stress states within zones of stress concentration, thereby increasing the risk of rock fracturing. Therefore, a thorough investigation into the mechanical properties of rocks under saturated conditions, as well as their deformation and fracturing characteristics, holds significant importance for the stability of underground rock and soil engineering.

Since the 1950s, with the acceleration of industrialization and urbanization processes, rock and soil engineering disasters induced by water–rock interactions have become increasingly common. Consequently, research on water–rock interactions has gradually emerged as a pivotal topic in the fields of rock mechanics and geotechnical engineering, primarily focusing on the influence of water on the physical–mechanical properties of rocks^1,2^. Vásárhelyi et al.^3^ through statistical analysis, observed a decrease in rock strength with increasing moisture content and concurrently developed strength and deformation evaluation criteria considering rock wet strength and water sensitivity. Vergara et al.^4^ through triaxial compression experiments, investigated the influence of moisture content on the mechanical properties of mudstone, revealing a high sensitivity of strength reduction within a certain range of saturation increase. With the advancement of technology, research on water–rock interactions has gradually expanded from the macroscopic to the microscopic scale. The application of techniques such as Scanning Electron Microscopy (SEM), numerical calculations, and Digital Image Correlation (DIC) has enabled researchers to explore the microscopic mechanisms of water–rock interactions in greater depth. Wu et al.^5^ conducted dry–wet cycling tests on 116 rock samples and summarized the mechanisms of sandstone weakening induced by dry–wet cycling as mineral expansion and contraction-induced and microcrack propagation, based on SEM image characteristics. Yu et al.^6^ using the MatDEM software, conducted immersion simulation tests on soft rocks, investigating the effects of fissures, clay mineral content, and initial moisture content on the softening process of rock samples.

Acoustic emission (AE) technology, by monitoring the elastic waves of rocks under stress, facilitates real-time tracking of crack propagation and initiation, providing a vital means to assess damage accumulation, prelude to fracture, and stability of rocks, thus becoming an indispensable component of research in water–rock interactions^7–9^. Zhao et al.^10^ based on the characteristic variations in AE event rates, categorized the deformation failure features of saturated sandstone into four stages, with the predominant mode of failure transitioning from tensile to shear failure. Cheng et al.^11^ suggested that the weakening of sandstone strength and AE signals primarily stem from the transformation of microcrack patterns induced by deteriorating mineral particle cementation. Furthermore, some researchers have, through the integration of frequency domain characteristics and fractal theory, unveiled the nonlinear dynamic features and self-organizing behavior during rock fracture processes. The application of machine learning techniques has further enhanced the ability to identify and predict rock failure processes^12,13^. By automating the analysis of AE data, machine learning can quickly recognize damage patterns and predict failure events. Methods such as Support Vector Machines (SVM), Neural Networks (NN), and Random Forests (RF) have been widely applied in the classification, regression, and pattern recognition of AE data. These methods can extract key features from complex AE signals, enabling the development of more accurate rock failure models. In recent years, the discovery of critical slowing down phenomena has shown significant potential in predicting rock failure^14–16^. Critical slowing down refers to discrete and fluctuating phenomena conducive to the formation of new phases near the critical point when the state of a complex dynamic system undergoes a transition^14,16,17^. However, current research on critical slowing down phenomena in the precursory stages of failure instability in saturated sandstone remains relatively limited. Hence, this study utilizes AE characteristic parameters to explore the critical slowing down features of saturated sandstone instability and failure during the loading process. Additionally, under external loading, the internal microstructure of rock undergoes changes, particularly the initiation and propagation of microcracks. These microcracks alter the electrical conduction pathways within the rock, leading to significant fluctuations in electrical resistivity^18–20^. By capturing these dynamic variations in resistivity, a novel perspective and potential approach for assessing rock damage and predicting rock failure are provided. Of course, other detection methods are also continuously being improved and are expected to be used in the monitoring and prediction of rock failure processes in the future^21–24^.

Currently, research on water–rock interactions has yielded substantial results, primarily focusing on the exploration of mechanical properties and physical characteristics. In practical deep subsurface geotechnical engineering, sandstone, as a common sedimentary rock, exhibits unique fracturing characteristics depending on its type. Based on previous studies, this paper conducts uniaxial compression acoustic emission (AE) experiments on four types of sandstone (green, white, brown, and red) and analyzes the mechanical properties and AE characteristics of saturated sandstone. Furthermore, by integrating RA–AF, resistivity changes, and critical slowing down theory, the paper delves into the fracturing characteristics and instability precursors of sandstone. The research findings not only enrich the theoretical framework for identifying fracture precursors in water-saturated sandstone but also provide important theoretical insights for the stability evaluation of deep geotechnical engineering projects.

## Materials and method

### Test material

**Fig. 1:**
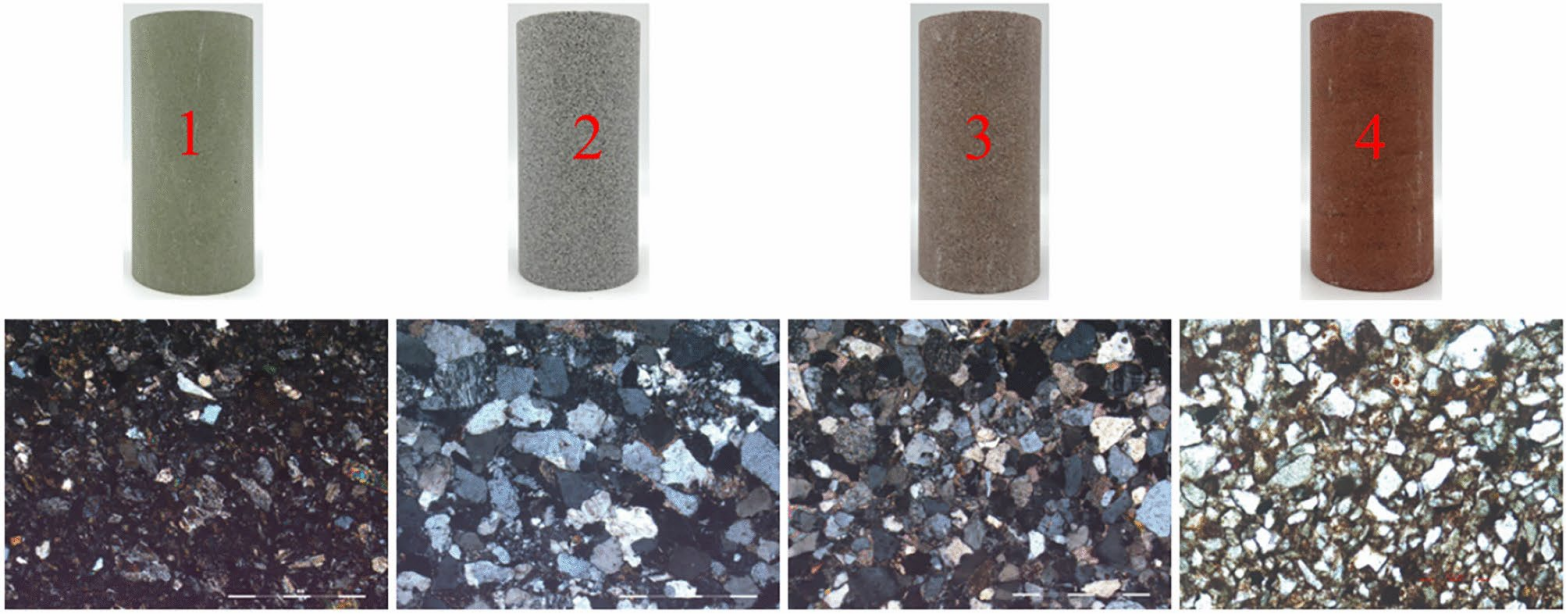
Sandstone samples.

For this experiment, a total of four distinct types of sandstone were selected, namely Shandong green sandstone, Guangxi white sandstone, Hunan brown sandstone, and Guizhou red sandstone. Following the requirements of international rock testing standards, the sandstone was fashioned into cylindrical specimens with a height-to-diameter ratio of 2:1 (height 100 mm, diameter 50 mm). To minimize the influence of end effects during testing, the parallelism of the end faces was controlled within a range of ± 0.02 mm. Subsequently, specimens with similar ultrasonic wave velocities were selected using a non-metallic ultrasonic wave velocity gauge for testing. Finally, thin sections of the samples were prepared, and thin section microscopic identification tests were conducted utilizing an Imager-type polarizing microscopy system (Fig. 1). The results revealed that the primary constituents of the sandstone include quartz, feldspar, mica, rock fragments, and void fillings. Of particular note is that the combined content of quartz, feldspar, and mica exceeds 88%, constituting the framework network of the sandstone (Table 1).

**Table 1:** Mineral composition percentage of sandstone.

Rock no	Feldspar/%	Quartz/%	Mica/%	Siliceous rock debris/%	Feldspar/%	Filler/%	Opaque mineral/%	Other/%
1	25	70	0.5	1	0.5	2	1	
2	30	60	0.5	3	0.5	4	1	1
3	25	66		3		5	0.5	0.5
4	40	48	1	1		5	4	1

### Test procedure and equipment

The processed samples of the four types of sandstone were divided into two groups: dry and saturated, with three samples in each group, totaling 24 specimens. Following the standards outlined in the “Engineering Rock Testing Methods,” the sandstone samples were initially placed in a drying oven at 105 degrees Celsius for 12 h to obtain dry sandstone. Subsequently, the samples were weighed, then immersed in a vacuum saturation device for 24 h to obtain saturated specimens. For ease of reference, the paper utilizes “Rock No. (1–4)” to respectively denote Shandong green sandstone, Guangxi white sandstone, Hunan brown sandstone, and Guizhou red sandstone. Additionally, the porosity of the sandstone was determined through nuclear magnetic resonance testing, with specific physical parameters of the rock samples detailed in Table 2.

**Table 2:** Physical parameters of different sandstones.

Rock No	Rocks	Dry mass/g	Saturated mass/g	Water absorption/%	Porosity/%	Dry p-wave/(m/s)	Saturated p-wave/(m/s)
1	Shandong green sandstone	513.24	517.05	0.73	1.731	3500	3200
2	Guangxi white sandstone	510.52	515.49	0.973	2.959	3600	3300
3	Hunan brown sandstone	475.51	488.67	2.767	7.762	3400	3100
4	Guizhou red sandstone	417.06	450.41	7.996	15.844	3300	3000

**Fig. 2:**
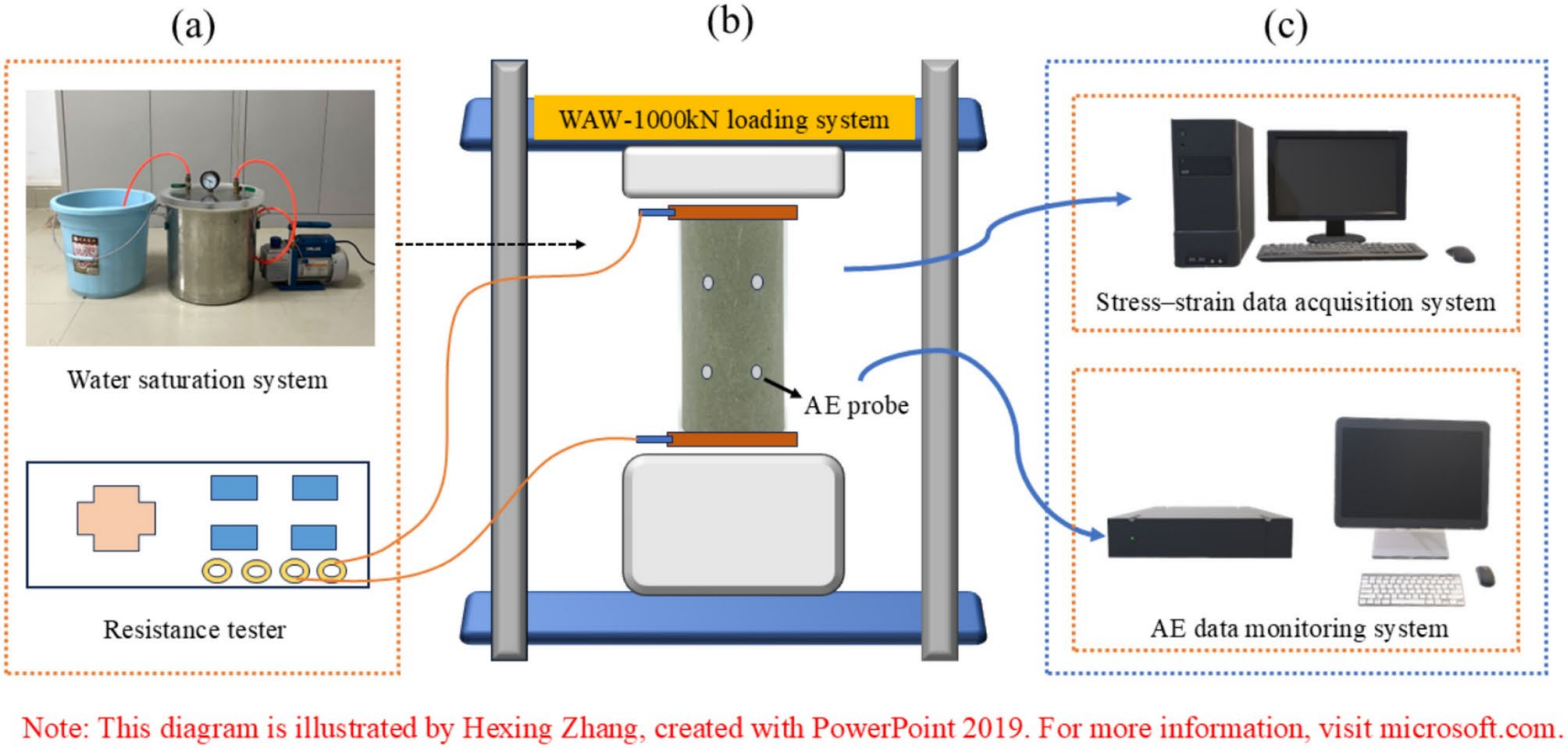
Test equipment.

The experimental setup comprises primarily a saturation system, stress loading monitoring system, Resistivity Tester, and AE signal acquisition system, as illustrated in Fig. 2. The WAW-1000kN rock mechanics system was employed in this study to conduct uniaxial compression tests on sandstone. The testing procedure utilized displacement control, with a loading rate set at 0.002 mm/s. Resistivity measurements were performed using the ANBO AT515 high-precision tester. The AE signals were collected using the PCI-2 system, with the NANO30 resonant sensor selected, featuring a center resonance frequency of 125 kHz. The pre-amplifier gain of the acoustic emission monitoring system was set to 40 dB, and the threshold for collecting ringing signals was set at 40 dB. An appropriate amount of coupling agent was applied between the specimen and the AE transducer to minimize noise.

## Results and analysis

### Softening characteristics of sandstone

**Fig. 3:**
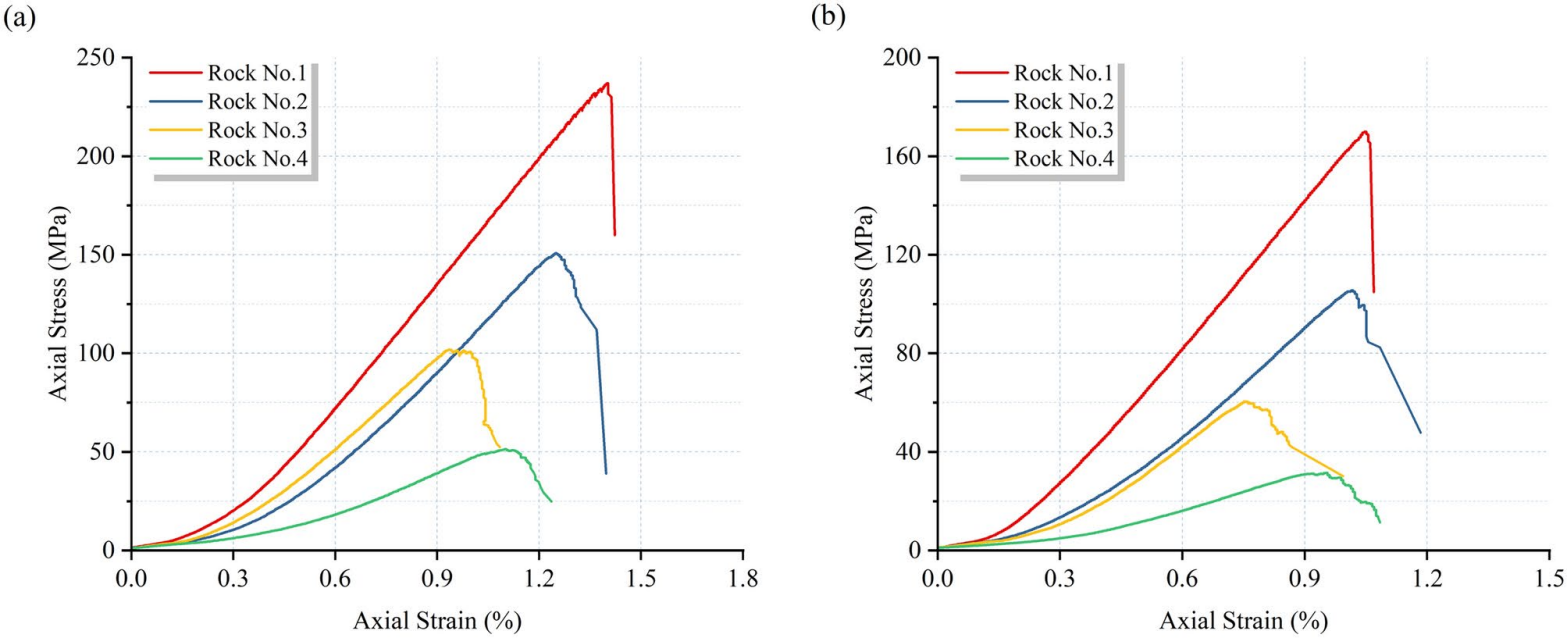
Stress–strain curves of sandstone: (**a**) dry state; (**b**) saturated state.

The stress–strain curve of the sandstone is depicted in Fig. 3. It is evident that the stress–strain curve of sandstone can be delineated into four distinct stages: compaction stage, elastic deformation stage, non-steady fracture development stage, and post-peak stage. Of particular note is the decrease in peak strain of saturated sandstone compared to its dry state. This phenomenon arises from the weakening of cohesion between mineral particles due to water molecules within the cracks, alongside the collective influence of pore water pressure, thereby diminishing the overall stiffness of the sandstone.

**Fig. 4:**
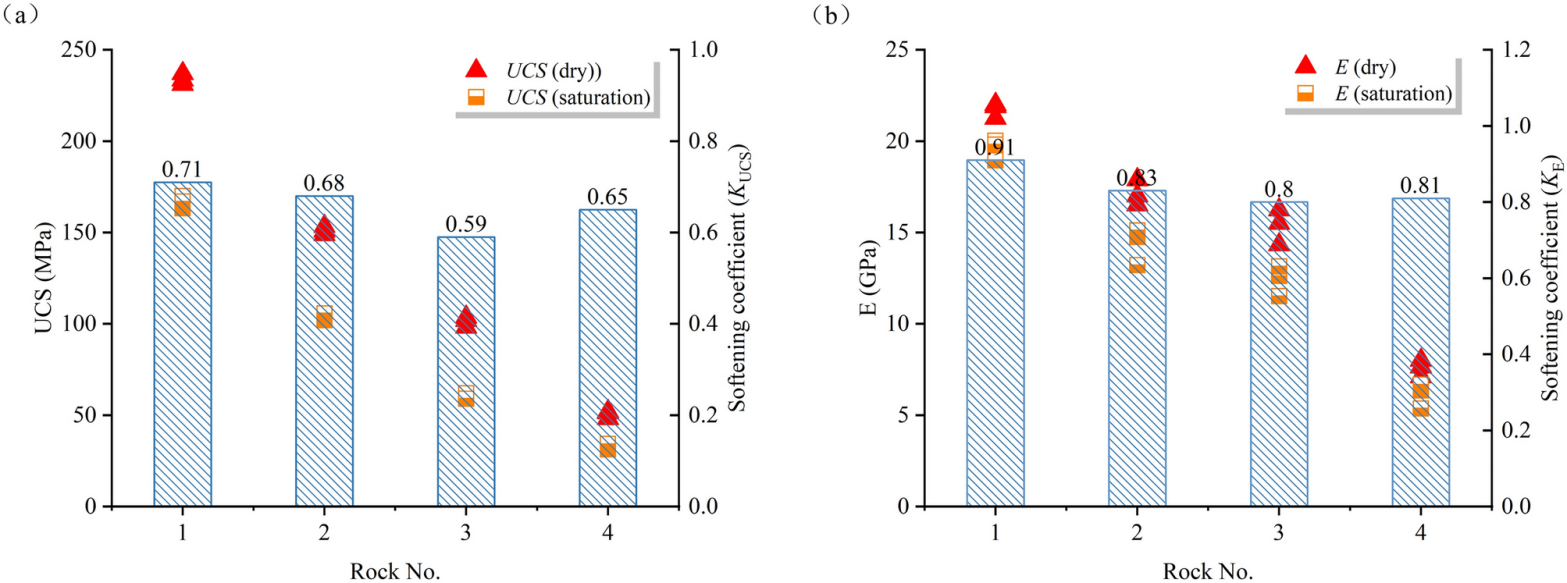
Mechanical properties and softening coefficient of sandstone: (**a**) UCS; (**b**) E.

By analyzing the stress–strain curves, the uniaxial compressive strength (UCS) and elastic modulus (E) of the sandstone were obtained, and further calculations were performed to determine its softening coefficient (K), as illustrated in Fig. 4. The softening coefficient is the ratio of mechanical parameters (UCS, E) under saturated conditions to those under dry conditions, denoted as K_UCS_ and K_E_, respectively. The UCS values for Rock No. (1–4) under dry and saturated conditions are 233.86, 151.01, 101.27, 50.52 MPa; 166.68, 103.9, 60.47, 33.03 MPa, respectively. Correspondingly, the E values are 21.71, 17.14, 15.36, 7.58 GPa; 19.79, 14.35, 12.42, 6.14 GPa, respectively. It is observed that the mechanical properties of saturated sandstone degrade significantly, with UCS and E decreasing by 28.72%, 31.19%, 40.28%, 34.62%; 8.84%, 16.27%, 19.14%, 18.99%, respectively. This indicates that water weakens the strength and deformation properties of the sandstone. The corresponding softening coefficients are 0.71, 0.68, 0.59, 0.65; 0.91, 0.83, 0.8, 0.81, respectively. A higher softening coefficient suggests a lesser degree of water-induced weakening of the rock’s mechanical properties, indicating a stronger resistance to water softening. Hence, the water softening performance of sandstone decreases sequentially from brown sandstone, red sandstone, white sandstone to green sandstone.

It is noteworthy that although No.1 has the lowest porosity and water absorption, it exhibits the greatest degree of softening in mechanical properties. This is primarily due to the high content of quartz (70%) and feldspar (25%), which may undergo hydration reactions in the presence of water, weakening the cementation between mineral particles and significantly reducing the rock strength. Additionally, the presence of mica (0.5%) and filler materials (2%) may induce microcrack propagation upon water absorption, further exacerbating the UCS softening effect of the sandstone.

### AE energy characterization of sandstone

**Fig. 5:**
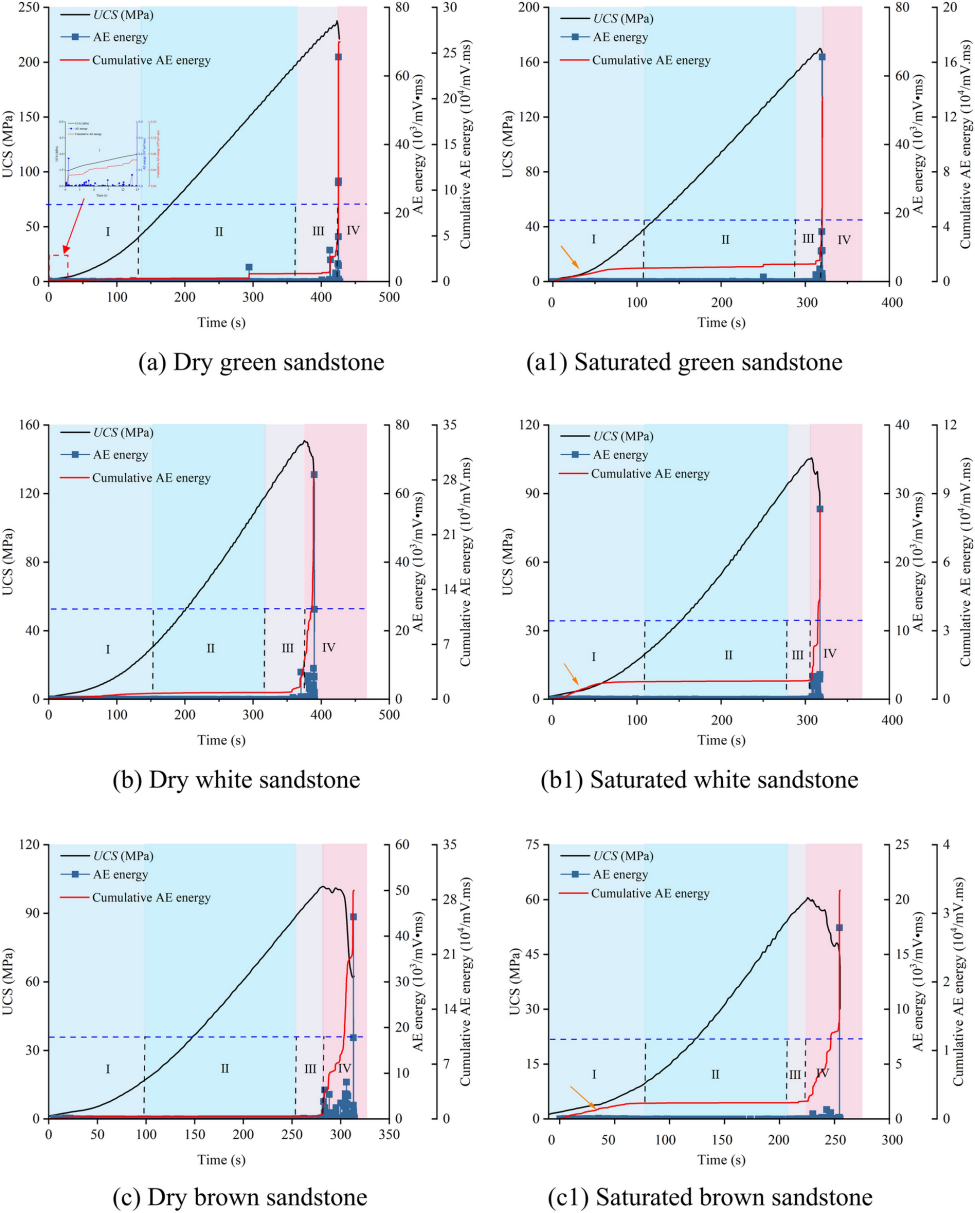

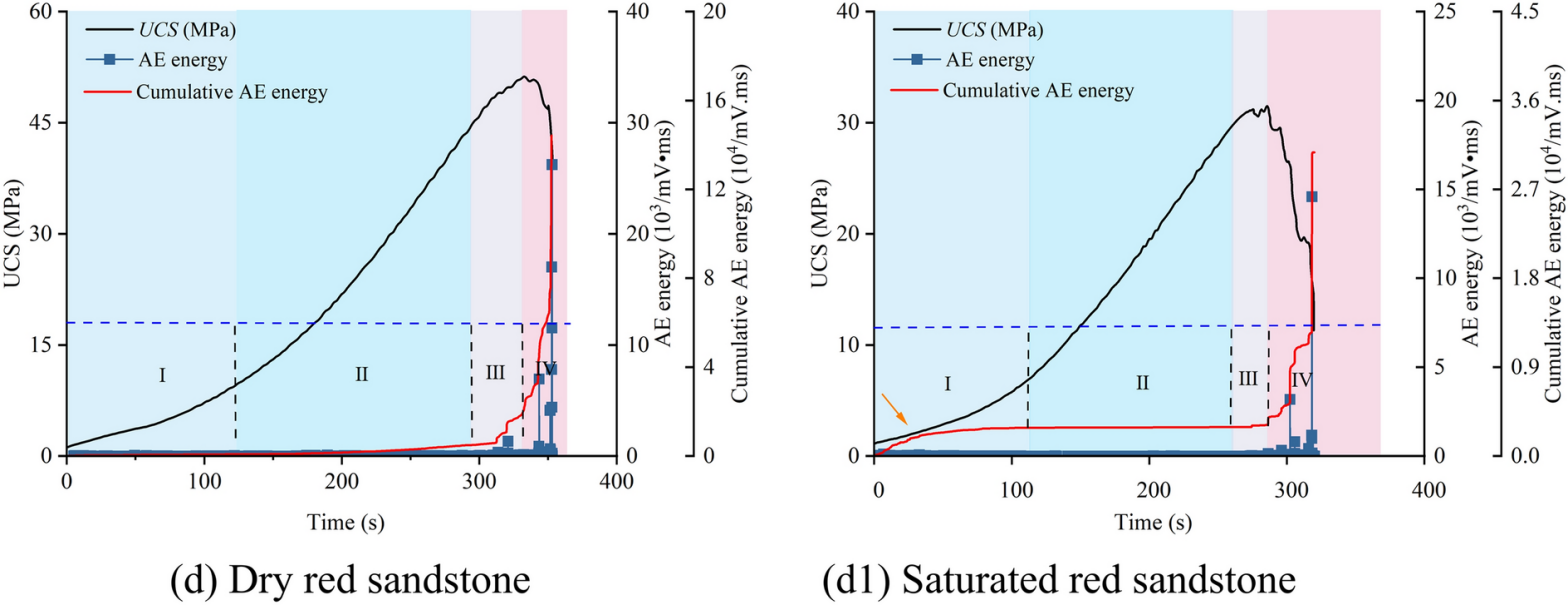
AE energy and cumulative AE energy characteristics of dry and saturated sandstones.

In this section, the fracture development trends of dry and saturated sandstone under uniaxial compression are analyzed based on the time-stress-AE energy-accumulated AE energy curves (Fig. 5).

The stress–strain curve of sandstone is delineated into four stages (Section “Softening characteristics of sandstone”). The cumulative AE energy curves of both dry and saturated sandstone exhibit similar patterns. Stage I: Within the natural rock samples, numerous initial microcracks and pores exist, which undergo compaction closure under stress. During this process, the rough surfaces on either side of the cracks are pressed together, leading to relative displacement of mineral particles. Accompanied by the release of a small amount of AE energy signals, the cumulative AE energy curve shows a slight increase (Fig. 5a). Stage II: During this stage, sandstone primarily undergoes elastic deformation, with minimal plastic damage. Due to relatively limited plastic deformation and damage activity, the cumulative AE energy curve remains relatively stable. Stage III: With the increase in stress, pre-existing cracks in the sandstone begin to propagate, accompanied by the appearance of new microcracks. Additionally, microcracks in some areas gradually nucleate, forming localized large-scale cracks. At this point, AE energy signals become active and dense, manifesting as a distinct stepped pattern in the cumulative AE energy curve. Stage IV: As microcracks intersect to form localized macroscopic cracks and connect to create primary cracks, fractures within the sandstone transition from local to macroscopic rupture surfaces, resulting in a sharp increase in the cumulative AE energy curve.

Although the AE energy and cumulative AE energy curves of dry and saturated sandstone exhibit similar trends, notable differences exist in their details. These disparities reflect the influence of water on the mechanical behavior and damage evolution of sandstone. Through statistical analysis, the cumulative AE energy values of Rock No. (1–4) in dry and saturated states are respectively: 26.21 × 10^4^, 29.03 × 10^4^, 29.2 × 10^4^, 14.42 × 10^4^ mV.ms; 13.46 × 10^4^, 8.29 × 10^4^, 3.33 × 10^4^, 3.07 × 10^4^ mV.ms. It is evident that the cumulative AE energy values of saturated sandstone experience significant reductions, representing 51.35%, 28.57%, 11.42%, and 21.28% of those observed in the dry state. This is primarily attributed to water ingress into the rock pore spaces, reducing the contact between mineral particles and softening clay minerals. Consequently, the degree of cementation between crystal particles weakens, thereby suppressing the release of AE energy. Furthermore, AE energy signals in saturated sandstone densely appear during the compaction stage, leading to a significant increase in cumulative AE energy values (Fig. 5a1–d1, indicated by yellow arrows). By separately calculating the proportion of cumulative AE energy values during the relatively stable stage (200 s) and at the point of failure for dry and saturated sandstone, the percentages are 1.38%, 2.8%, 1.22%, and 1.06%; 7.8%, 9.47%, 7.09%, and 9.48%, respectively. This phenomenon arises from the reduction in rock volume and compression of pore water during the compaction stage, resulting in increased pore water pressure, which, in turn, promotes the expansion and connection of microcracks. At this stage, AE energy signals appear more densely, leading to a significant increase in cumulative AE energy values.

The underlying cause of macroscopic failure in rocks under external loading is the accumulation of damage, wherein the magnitude of cumulative AE energy serves as a measure of the rock’s accumulated damage under stress^25,26^. From the characteristics of cumulative AE energy, it is evident that the presence of water significantly weakens the cumulative damage effect in sandstone. Drawing upon the aforementioned analysis, the degree to which the cumulative damage is influenced by water follows the order: brown sandstone, red sandstone, white sandstone, and green sandstone.

### Resistivity characteristics of sandstone during uniaxial loading

The resistivity of the sandstone specimen is calculated using the following formula:

1 $$\rho = R\frac{S}{L}$$

where: $\rho$ is the resistivity, $R$ is the resistance (Ω), $S$ is the cross-sectional area of the specimen (m^2^), L is the length of the specimen (m).

**Fig. 6:**
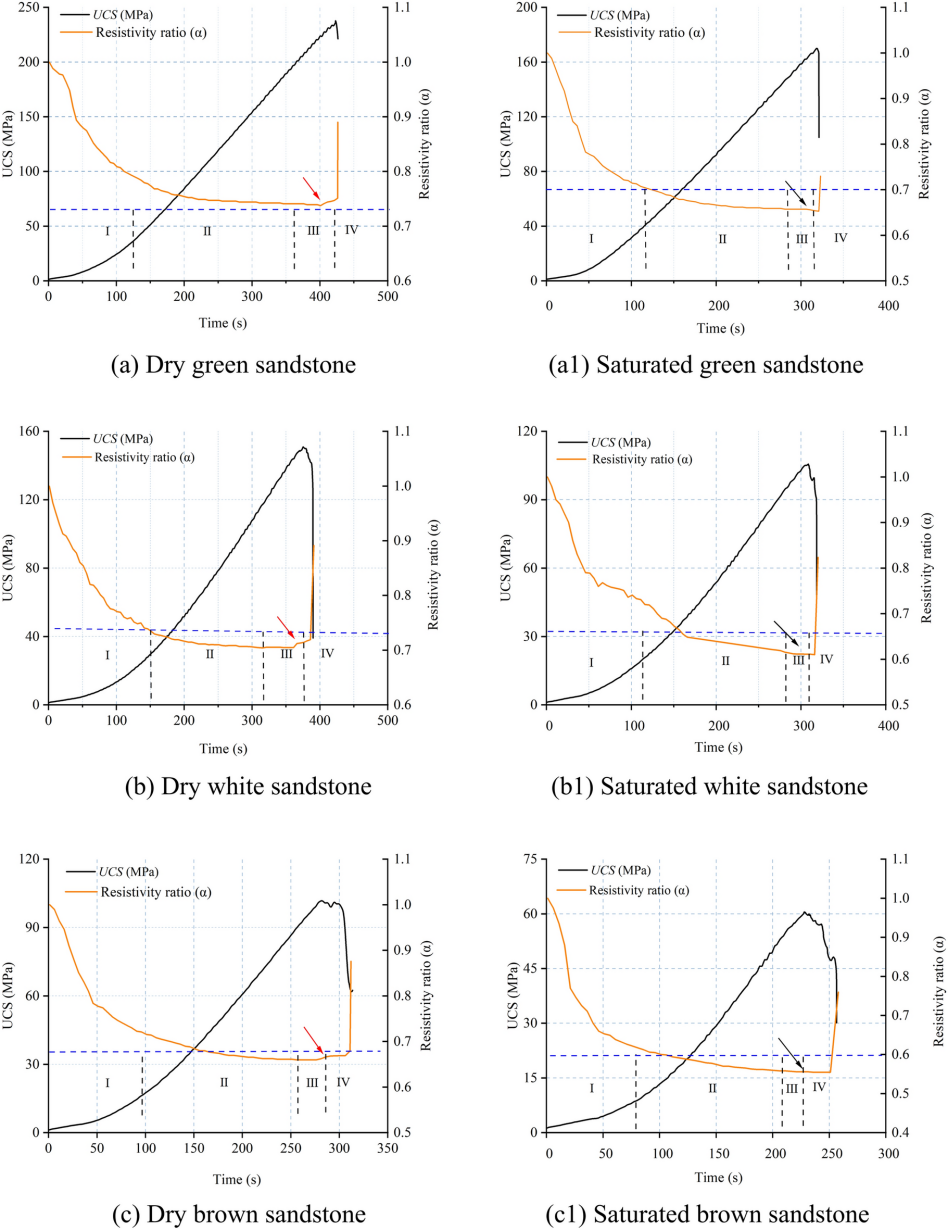

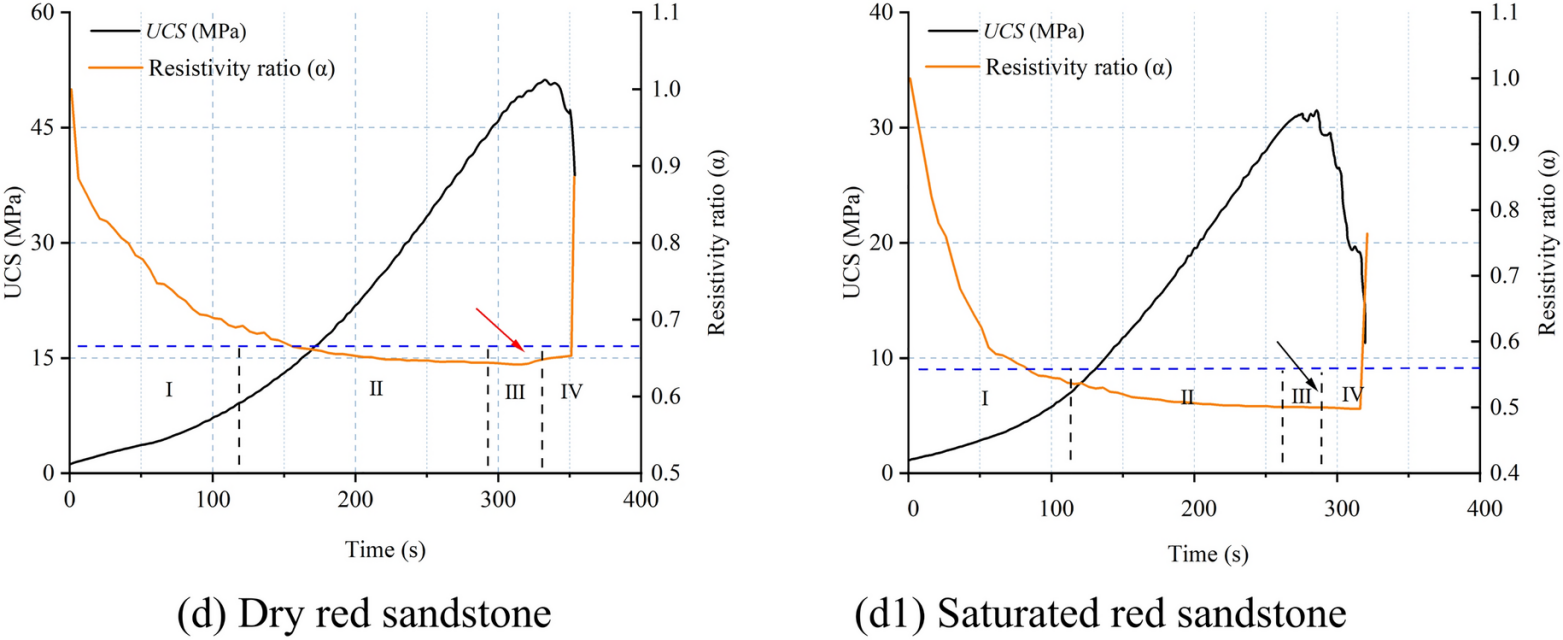
Resistivity ratio variation curves for dry and saturated sandstones.

To simplify the analysis of the resistance change with loading, the parameter α, the resistivity ratio (the ratio of real-time resistivity to initial resistivity), was introduced. Figure 6 shows the resistivity ratio curves for the sandstones. The resistivity trends of the four types of sandstone exhibit consistency. Stage I: The initial microcracks and pores within the sandstone begin to close under low pressure, increasing the contact area between mineral grains. This improves the overall conductivity of the sandstone, causing the resistivity of both dry and saturated samples to decrease rapidly. Stage II: As stress gradually increases, the sandstone framework becomes more compact, and the resistivity decreases slowly. Stage III: The interaction of primary microcracks leads to the initiation, propagation, and coalescence of secondary cracks. For dry sandstone, conductivity decreases due to the destruction of the mineral framework, with resistivity first decreasing and then increasing (red arrow). In saturated sandstone, the formation of interconnected cracks leads to the rapid development of a water film, resulting in a secondary decrease in resistivity (black arrow). Stage IV: The fractures in the sandstone propagate along macroscopic cracks, disrupting the conductive pathways, which causes a significant increase in resistivity for both dry and saturated samples.

In the dry state, the resistivity of the four sandstones decreased to 73.7%, 70.4%, 65.9%, and 64.2% of their initial values, respectively. In the saturated state, these percentages were 65.3%, 59.9%, 55.4%, and 49.7%. This indicates that saturated sandstone is more sensitive to resistivity changes due to internal fracturing. Porosity has a significant direct impact on resistivity; the higher the porosity, the greater the decrease in resistivity. By analyzing the characteristics of the AE energy curves, it was observed that in the early stages of the uniaxial compression test (Stages I and II), resistivity changes showed high sensitivity. In contrast, in the later stages (Stages III and IV), the increase in AE energy was more pronounced. This indicates that resistivity and AE monitoring have different sensitivities to internal damage in sandstone, and they exhibit strong regularity and complementarity.

### Analysis of sandstone failure behavior based on RA–AF

#### Distributional characteristics of RA–AF

**Fig. 7:**
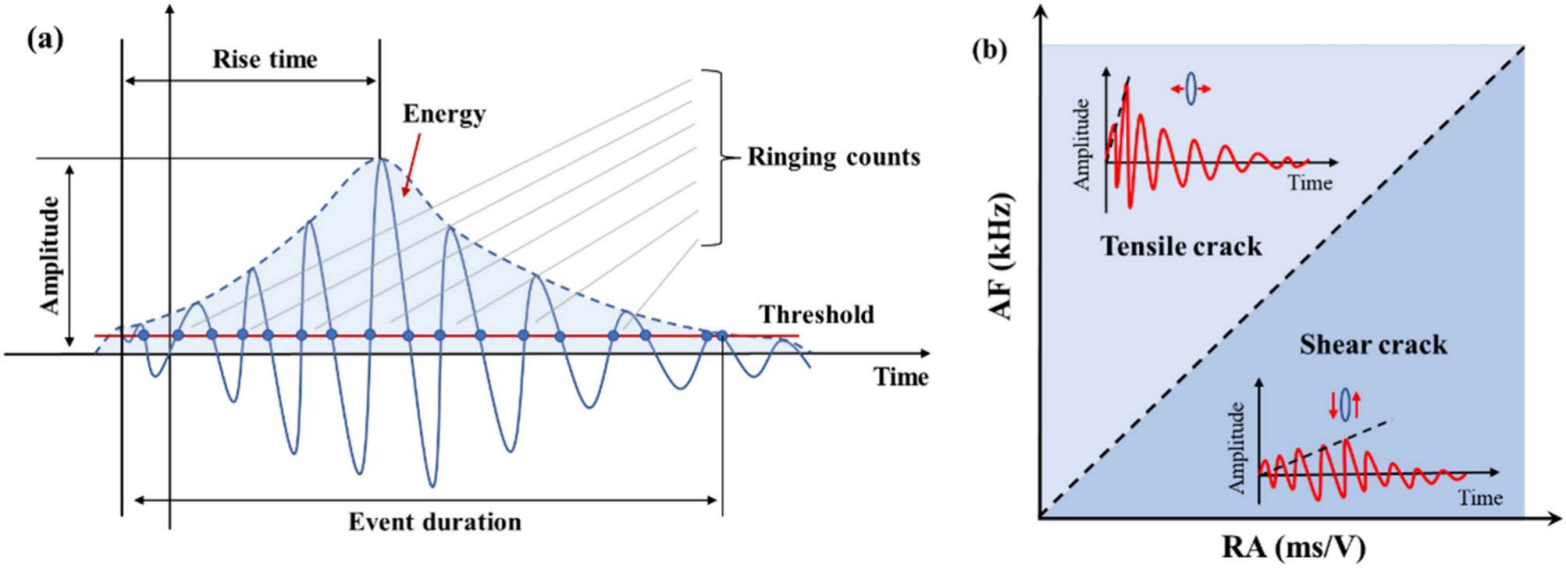
Schematic diagram of RA–AF^28^.

In rock mechanics research, parameters like the rise time-to-amplitude ratio (RA) and average frequency (AF) are commonly used to analyze crack propagation mechanisms. This approach was first proposed by the Japan Concrete Institute in 2003, linking AF and RA to evaluate the cracking failure mechanism of concrete materials (JCMS-III B5706, 2003)^27^. The calculation methods for RA and AF are as follows:

2 $$RA = \frac{{A_{RT} }}{{A_{A} }}$$

3 $$AF = \frac{{A_{C} }}{{A_{D} }}$$

where ${A}_{RT}$ denotes the rise time, ${A}_{A}$ signifies the amplitude; ${A}_{C}$ stands for the ringing count, and ${A}_{D}$ represents the duration. Typically, low values of AF and high values of RA indicate the generation and evolution of shear cracks, whereas high AF values and low RA values indicate the generation and evolution of tensile cracks (Fig. 7).

**Fig. 8:**
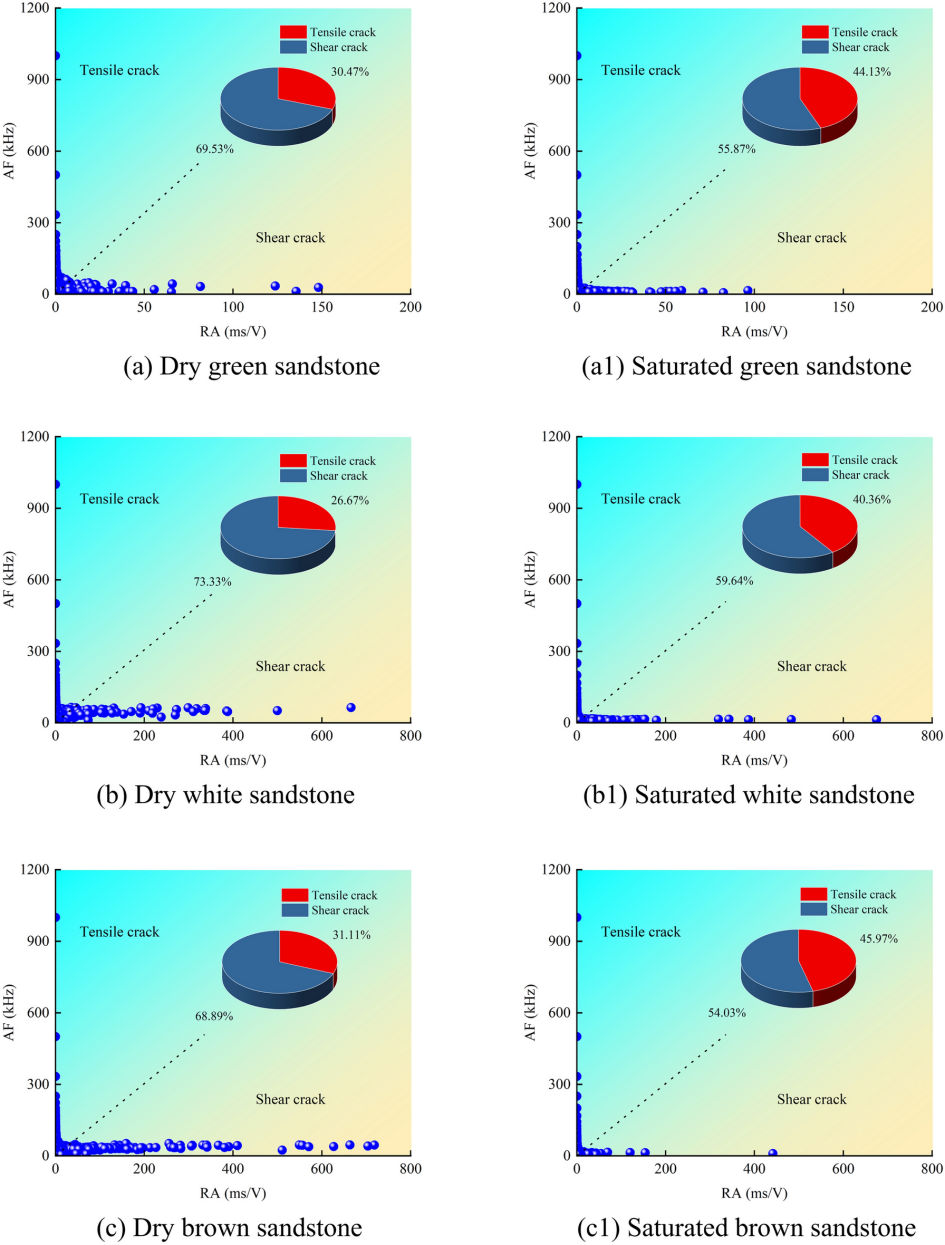

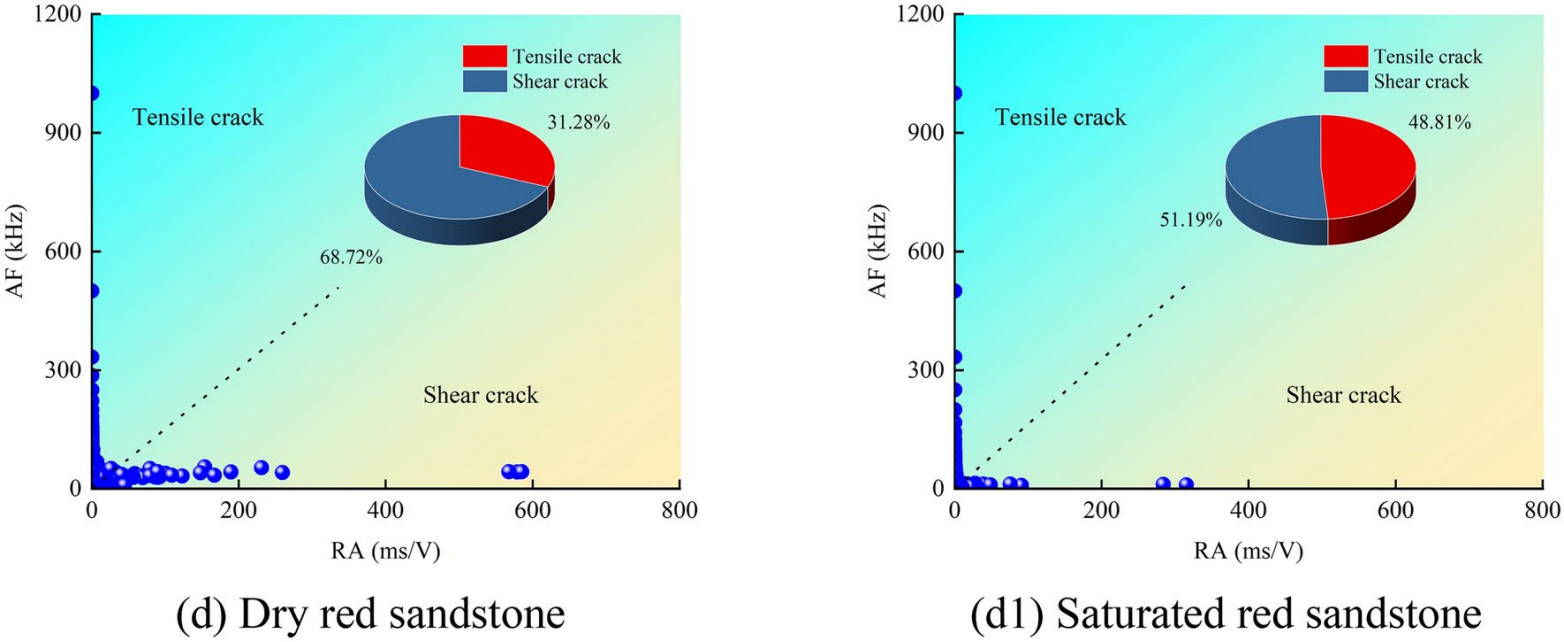
RA–AF scatter distributions for dry and saturated sandstones.

**Fig. 9:**
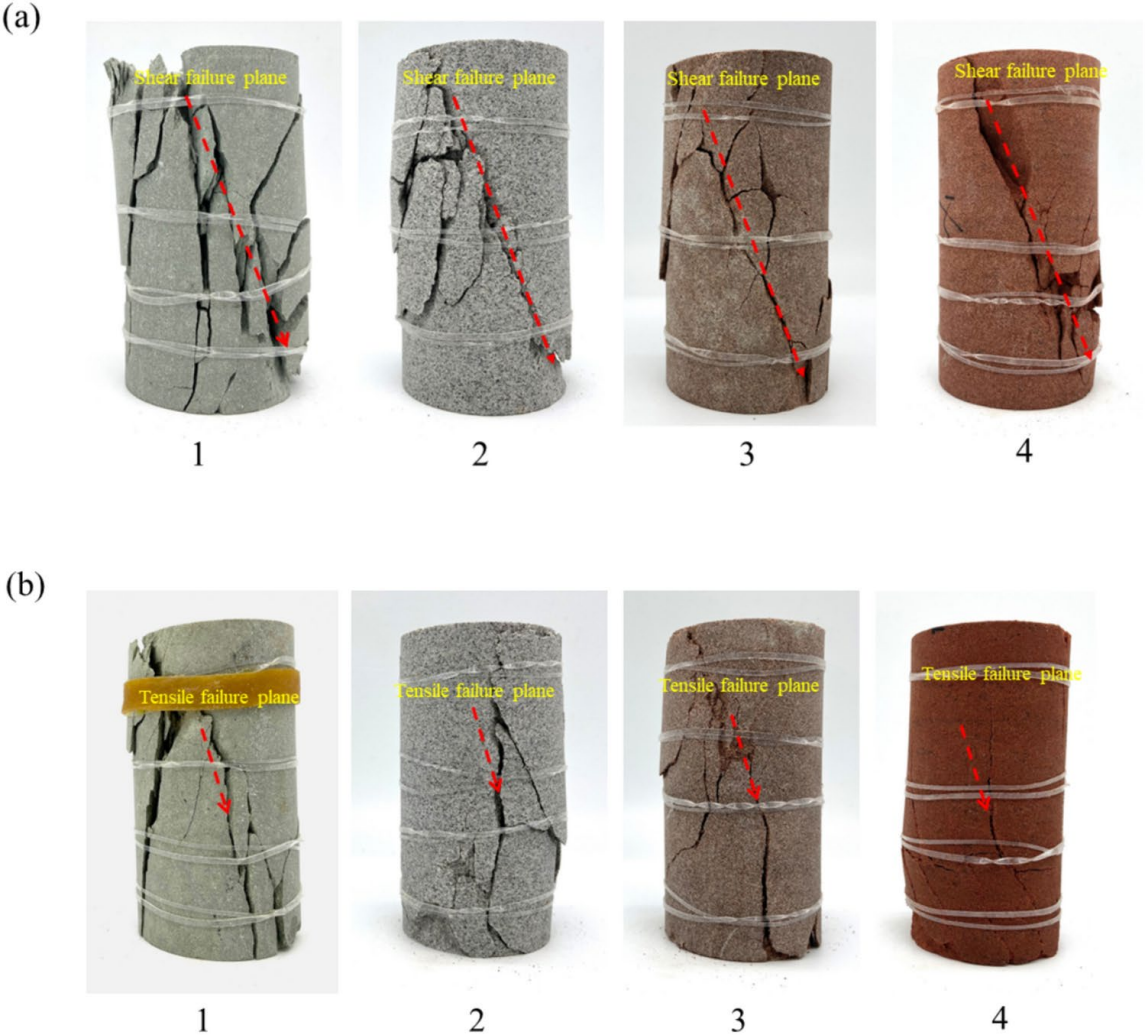
Failure mode of sandstone: (**a**) dry; (**b**) saturated.

Utilizing the aforementioned definitions, the RA and AF values of sandstone under uniaxial compression are computed, and the corresponding scatter plots are depicted in Fig. 8. It is discernible that the AF values of sandstone primarily range between 0 and 1200 kHz, while the RA values predominantly span from 0 to 800 ms/V. Notably, the region of shear cracks exhibits the most extensive distribution of RA–AF signals, with the densest concentration near the origin (0 –200 ms/V), gradually thinning out with increasing distance from the origin (200–800 ms/V). It is noteworthy that the RA values of shear cracks in dry sandstone exhibit a wider distribution range and a greater quantity (Fig. 8a–d). Conversely, the distribution range and quantity of RA values in saturated sandstone noticeably diminish and converge toward the origin (Fig. 8a1–d1). By evaluating the slope (AF = 200) of the RA/AF linear line to distinguish between tensile and shear cracks, the proportional distribution of different types of cracks is determined^29,30^. In dry sandstone, the proportions of tensile and shear cracks are as follows: 30.47%, 26.67%, 31.11%, 31.28%; 69.53%, 73.33%, 68.89%, 68.72%, respectively. Conversely, under saturated conditions, these proportions are: 44.13%, 40.36%, 45.97%, 48.81%; 55.87%, 59.64%, 54.03%, 51.19%. Overall, both dry and saturated sandstones are predominantly characterized by shear cracks, yet the presence of water has led to a respective increase of 13.66%, 13.69%, 14.86%, 17.53% in tensile cracks in the water-bearing sandstone. Particularly, the high porosity of red sandstone provides favorable conditions for pore water pressure, leading to a notable increase in tensile crack growth. In summary, the presence of water promotes the initiation of tensile cracks while inhibiting the development of shear cracks. Furthermore, the proportions of cracks classified based on RA–AF correspond to the macroscopic failure morphology (Fig. 9), notably, an increase in tensile failure surfaces on the surface of fully saturated sandstone (Fig. 9b).

#### Cumulative crack and energy curve characteristics

**Fig. 10:**
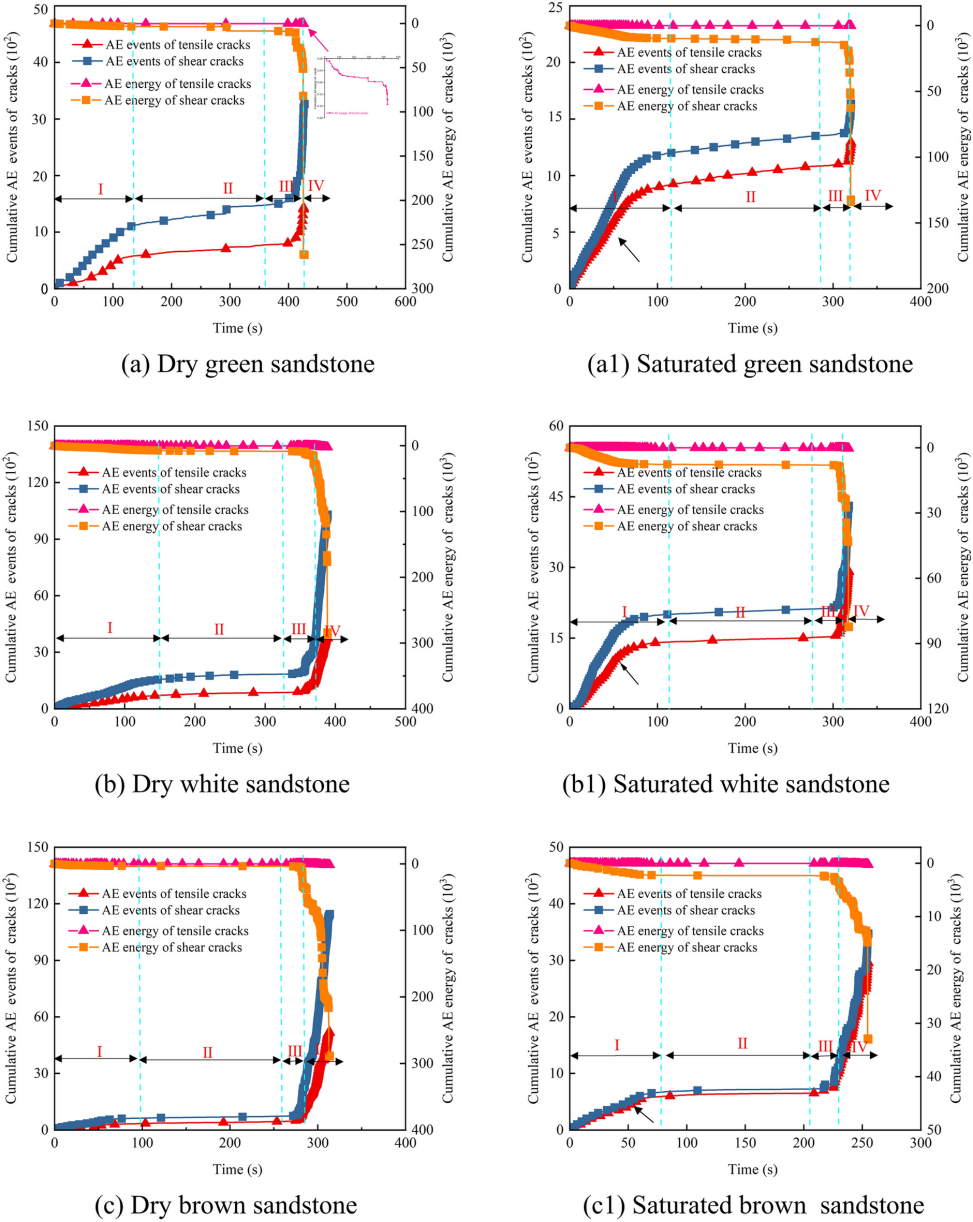

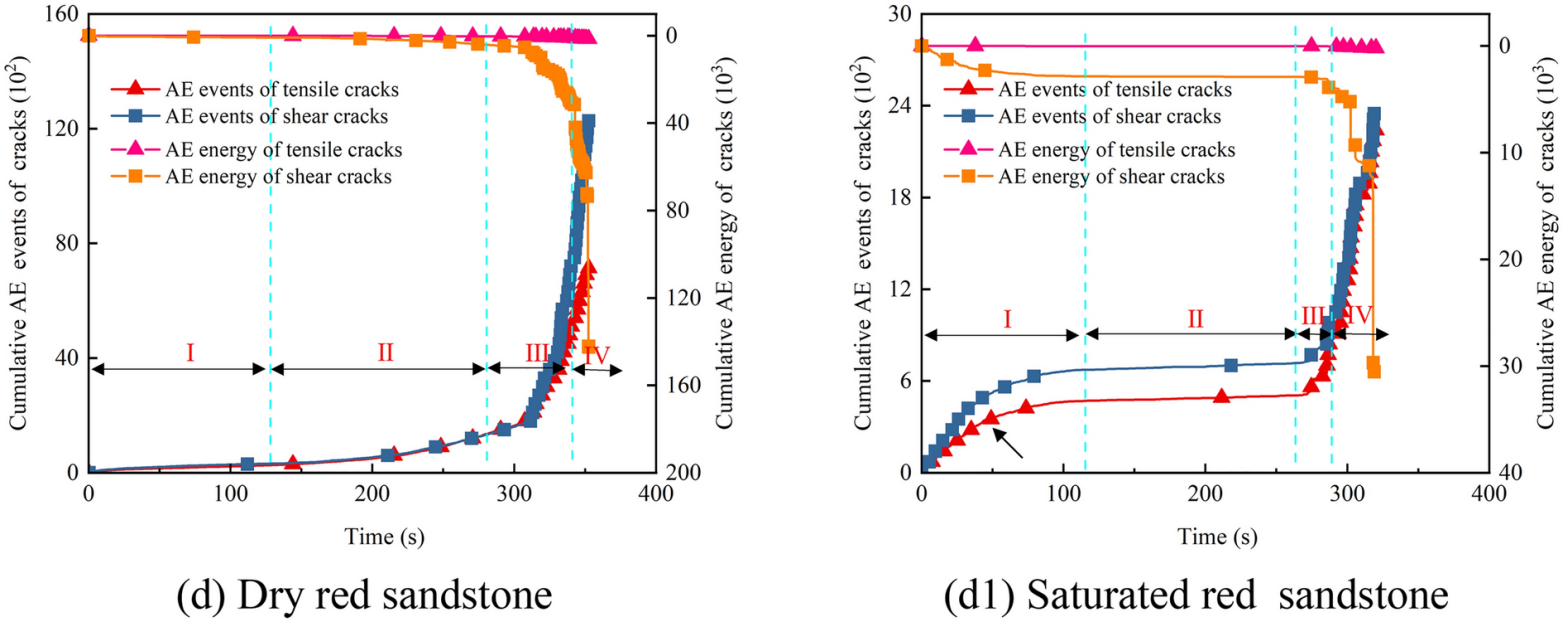
Cracking and AE energy evolution patterns in dry and saturated sandstones.

As illustrated in Fig. 10, the cumulative tensile and shear crack curves of sandstone under varying moisture conditions exhibit four discernible stages: gradual ascent, stabilization, periodic ascent, and rapid escalation. Particularly near the stress peak, there is a pronounced increase in crack propagation. It is noteworthy that the trends in cumulative crack curves mirror those of cumulative AE energy curves, as depicted in Fig. 5. This alignment underscores the efficacy of AE energy signals in reflecting internal fracture tendencies within the sandstone. During the loading process of dry sandstone, both tensile and shear cracks emerge simultaneously, displaying similar trends (Fig. 10a–d). Specifically, in Stage I, a small number of cracks appear; in Stage II, an extended period of quiescence ensues; starting from Stage III, crack propagation resumes; in Stage IV, crack proliferation accelerates sharply, reaching a peak in the cumulative curve. Notably, the cumulative count of shear cracks consistently surpasses that of tensile cracks, with Stage IV exhibiting the most pronounced difference, mirroring the macroscopic failure pattern. Under saturated conditions, the trend in cumulative crack curves resembles that observed during dry conditions. However, there is a rapid increase in the number of tensile cracks at the onset of loading (Fig. 10a1–d1, indicated by the black arrows). This acceleration is attributed to the influence of pore water pressure, which promotes the initiation and propagation of tensile cracks.

**Fig. 11:**
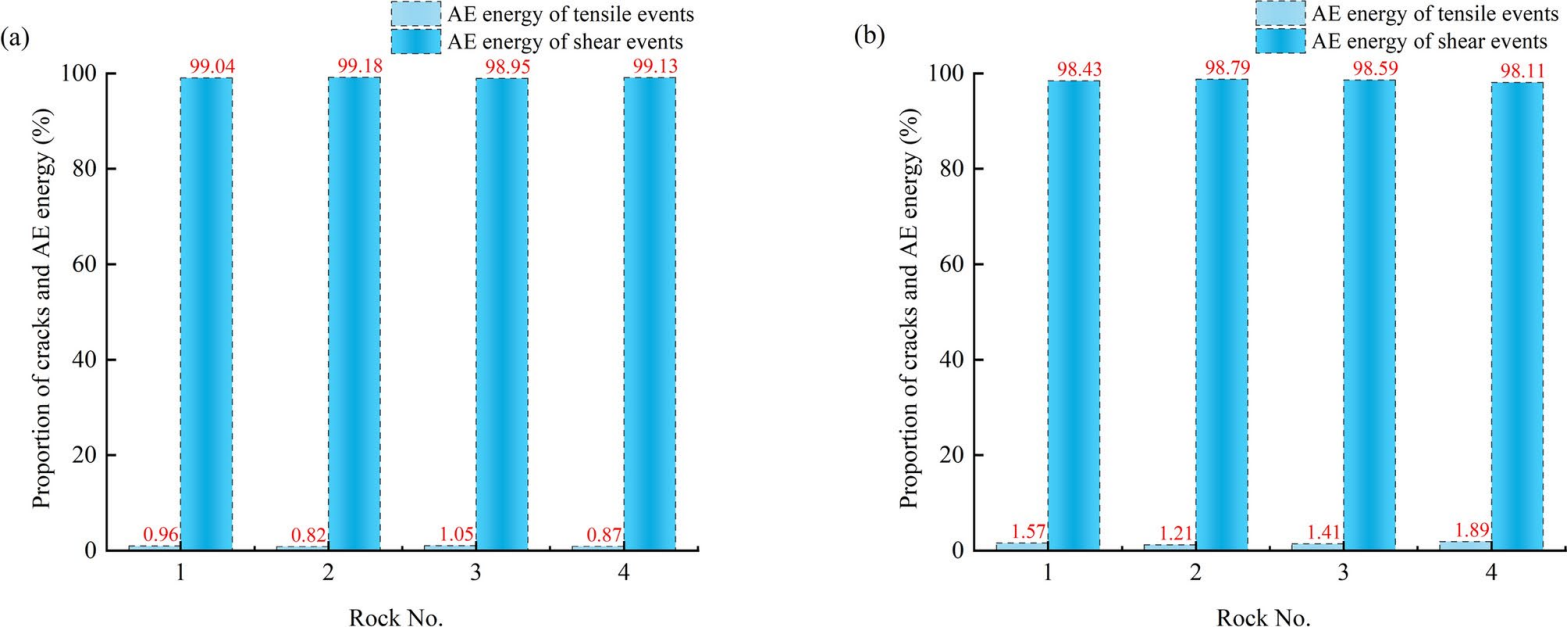
AE energy ratios for sandstone cracks: (**a**) Dry; (**b**) Saturated.

Moreover, the cumulative shear crack curve aligns with the corresponding cumulative energy trend. Due to the relatively modest cumulative AE energy associated with tensile cracks, they exhibit a linear trend when contrasted with the AE energy of shear cracks. A closer examination through the localized magnification in Fig. 10a allows for clear observation of the consistent cumulative AE energy trends for both types of cracks. As depicted in Fig. 11, we computed the proportion of AE energy attributed to cracks. In dry sandstone, the AE energy for tensile and shear cracks respectively amounts to: 0.96%, 0.82%, 1.05%, 0.87%; and 99.04%, 99.18%, 98.15%, 99.13%. Conversely, under saturated conditions, these proportions stand at: 1.57%, 1.21%, 1.41%, 1.89%; and 98.43%, 98.79%, 98.59%, 98.11%. It is evident that shear cracks in sandstone command an absolute dominance in cumulative AE energy, accounting for over 98.11% in all cases. Despite a notable increase of over 13% in the number of tensile cracks under saturated conditions, the corresponding AE energy only marginally rises by 0.4%. Notably, red sandstone exhibits the greatest increments in both the number and energy of tensile cracks, possibly attributed to variations in porosity. It is worth highlighting that while the cumulative AE energy for both types of cracks approaches similarity in Stages I, II, and III, a significant disparity emerges in Stage IV. Combining this observation with the characteristics of cumulative crack curves indicates that this discrepancy arises from extensive shear slip and dislocation of micro-cracks during this stage, leading to the formation of macroscopic shear planes and the release of additional AE energy.

Synthesizing the above analysis reveals that the cumulative number of shear cracks in sandstone consistently exceeds that of tensile cracks, aligning with the macroscopic failure morphology. Under saturated conditions, despite the substantial generation of tensile cracks facilitated by pore water pressure, the predominant mode of macroscopic failure remains shear, accompanied by a comparatively minor increase in corresponding AE energy.

### Critical slowing down characteristics of sandstones

Through an in-depth analysis of the AE signals during uniaxial loading, we have observed several characteristic parameters undergoing significant changes approaching the macroscopic failure stage, exhibiting pronounced fluctuating distributions. This phenomenon is commonly referred to as critical slowing down. Hence, computing and analyzing the variance and autocorrelation coefficients of AE characteristic parameters over time can offer a precursor prediction method for macroscopic failure in rock mechanics^31^.

The variance is a measure of the extent to which data in a sample deviate from the mean and can be expressed as:

4 $${S}^{2}=\frac{1}{n}\sum_{i}^{n} ({x}_{i}-\overline{x }{)}^{2}$$

where: ${\text{S}}^{2}$ is the variance, n is the total number of sample data, x_i_ is the ith data, $\overline{\text{x} }$ is the mean. The autocorrelation coefficient is a statistical measure of the correlation between the same variable at different time intervals. The autocorrelation coefficient with a lag length of j for variable x can be expressed as:

5 $$a\left( j \right) = \sum\limits_{i = 1}^{n - j} {\left( {\frac{{x_{i} - \overline{x}}}{s}} \right)\left( {\frac{{x_{i + j} - \overline{x}}}{s}} \right)}$$

where: s is the mean square deviation. Assuming that there is a forced perturbation of the state variable with period $\Delta t$, during the perturbation, the rate of recovery is $\lambda$, an approximate exponential relationship, which can be described in the regression model as:

6 $${y}_{n+1}={\text{e}}^{\lambda\Delta t}{y}_{n}+s{\varepsilon }_{n}$$

where: ${y}_{n}$ is the deviation of the system variable from equilibrium and ${\varepsilon }_{n}$ is a random quantity that conforms to a normal distribution. If $\lambda$ does not depend on ${y}_{n}$, the process can be simplified as follows:

7 $${y}_{n+1}=a{y}_{n}+s{\varepsilon }_{n}$$

where: a is the autocorrelation coefficient, $a={e}^{\lambda\Delta t}$. Analysis of Eq. (8) by variance Var yields:

8 $$Var({y}_{n+1})=\text{E}({y}_{n}^{2})+[\text{E}({y}_{n}){]}^{2}=\frac{{s}^{2}}{1-{a}^{2}}$$

where: E is the mathematical expectation. As the system progressively approaches the critical point, the response to minor perturbations gradually slows down, and the recovery rate $\lambda$ eventually approaches 0. Concurrently, the system’s autocorrelation coefficient $a$ tends towards unity, while the variance approaches infinity. Hence, the sudden increase in variance and autocorrelation coefficient serves as a precursor signal indicating the system’s approach to the critical point.

In this section, we will employ parameters that maintain the integrity of the time series for variance and autocorrelation coefficient analysis, such as amplitude, RA value, rise time, and AE energy. Scholarly validation has indicated that the window length and lag step have minimal impact on the timing of precursor points for rock instability failure and can be disregarded^32,33^. Thus, we adopt a window length of 200 and a lag step of 2 to compute the time series of variance and autocorrelation coefficients for four AE parameters, analyzing their variations, with a focus on their abrupt changes nearing failure. We aim to explore whether the autocorrelation coefficients and variances of these parameters can serve as precursor information for the transition from rock damage fracture to instability failure based on critical slowing down theory conditions.

#### Autocorrelation coefficient characteristics of AE parameters

**Fig. 12:**
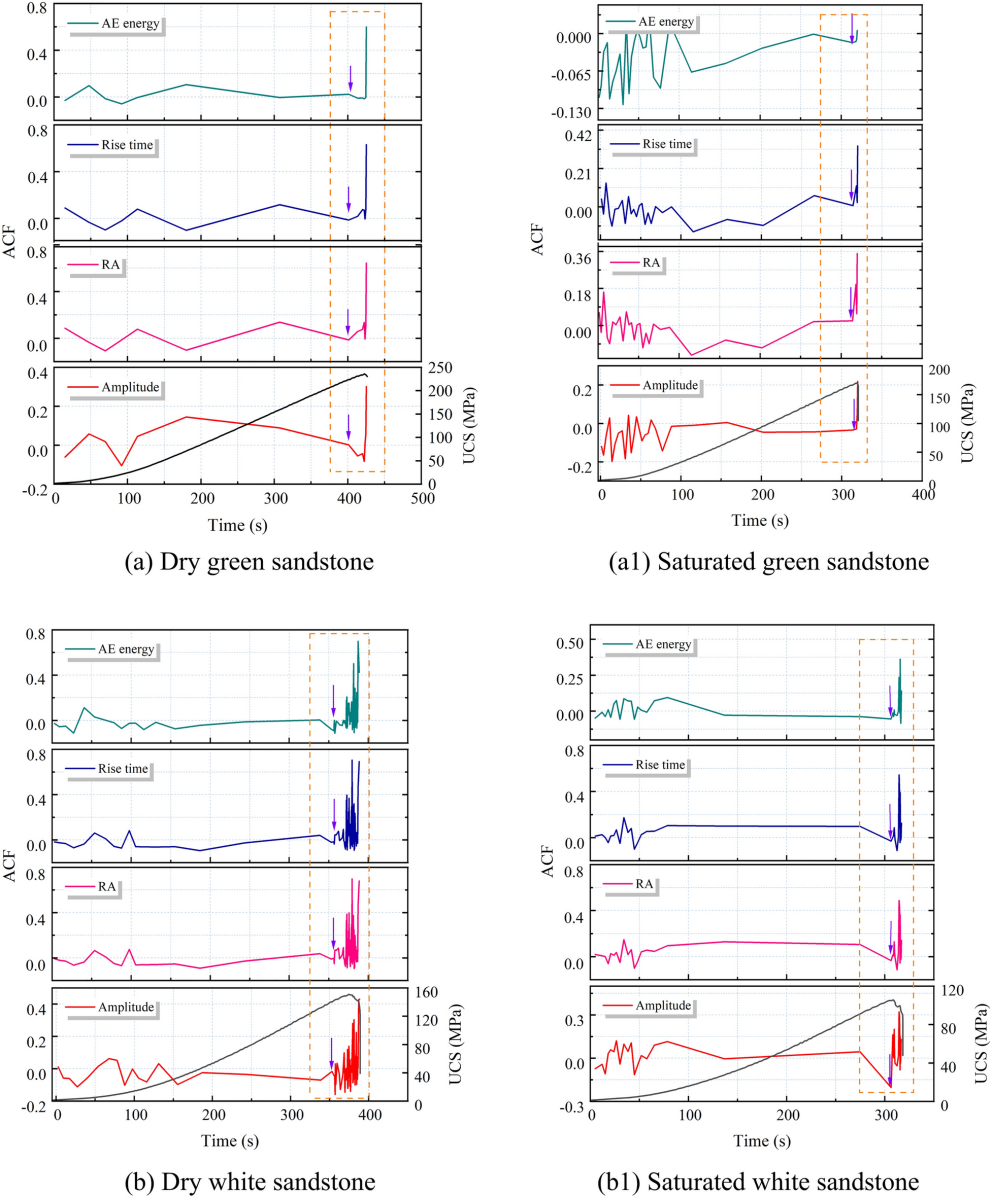

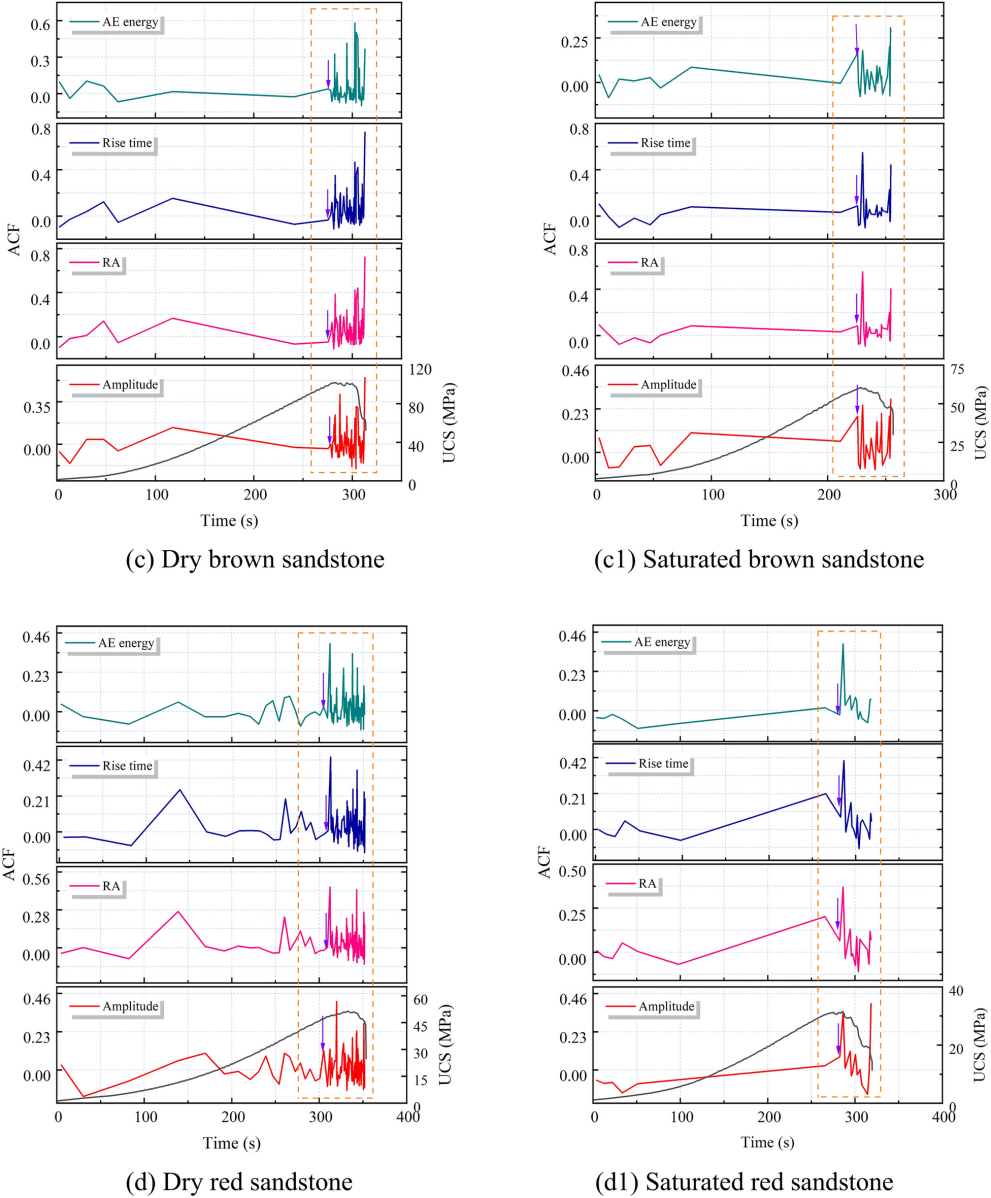
Autocorrelation coefficient pattern of AE parameters for dry and saturated sandstone.

The autocorrelation coefficient trends of four AE parameters were calculated based on critical slowing down theory, as depicted in Fig. 12. It can be observed that the autocorrelation coefficients of amplitude, RA value, rise time, and AE energy remain relatively stable during the loading process but exhibit a sharp increase nearing failure. This indicates a pronounced critical slowing down phenomenon in these AE parameters, serving as indicators of precursor characteristics for rock failure. Moreover, the autocorrelation coefficient curves of the four AE parameters demonstrate similar trends and shapes, with highly coincident onset points on the time scale, suggesting equal sensitivity and reliability as early warning indicators. Notably, fluctuations in the autocorrelation coefficients occur during the compaction and elastic stages. This is attributed to minor crack propagation causing subtle changes in the system phase state, yet the system can quickly return to a stable state. In comparison to the dry state, the autocorrelation coefficients of saturated sandstone exhibit an increased amplitude of fluctuations during the early loading stage, indicating an escalated level of internal damage and fracture at this phase. This shift is attributed to the effect of pore water pressure, which facilitates the expansion and development of cracks, aligning with the findings of section “Distributional characteristics of RA–AF”. Additionally, nearing failure, the autocorrelation coefficients of AE parameters in dry sandstone exhibit dense fluctuations, which diminish in intensity under saturated conditions. This phenomenon arises from the lubricating action of water within the sandstone, mitigating friction among internal particles and thus attenuating the system’s phase state transition.

From the foregoing analysis, it is evident that the presence of water induces variations in the autocorrelation coefficient characteristics of sandstone. A careful examination reveals that these characteristics are concurrently influenced by the type of sandstone. Notably, the abrupt increase in autocorrelation coefficients is observed in green sandstone, whereas red sandstone exhibits multiple pseudo-signals prior to failure, and other sandstones display dense fluctuations only nearing failure. This disparity arises because sandstones with lower porosity feature a more uniform microstructure, leading to synchronized and concentrated stress concentration and crack propagation before failure, thereby causing a sudden change in the system’s phase state. Conversely, sandstones with higher porosity possess a complex microstructure, resulting in crack propagation and stress concentration occurring at different times and locations, leading to a gradual and fluctuating process in the system’s phase state approaching critical conditions.

#### Variance characteristics of AE parameters

**Fig. 13:**
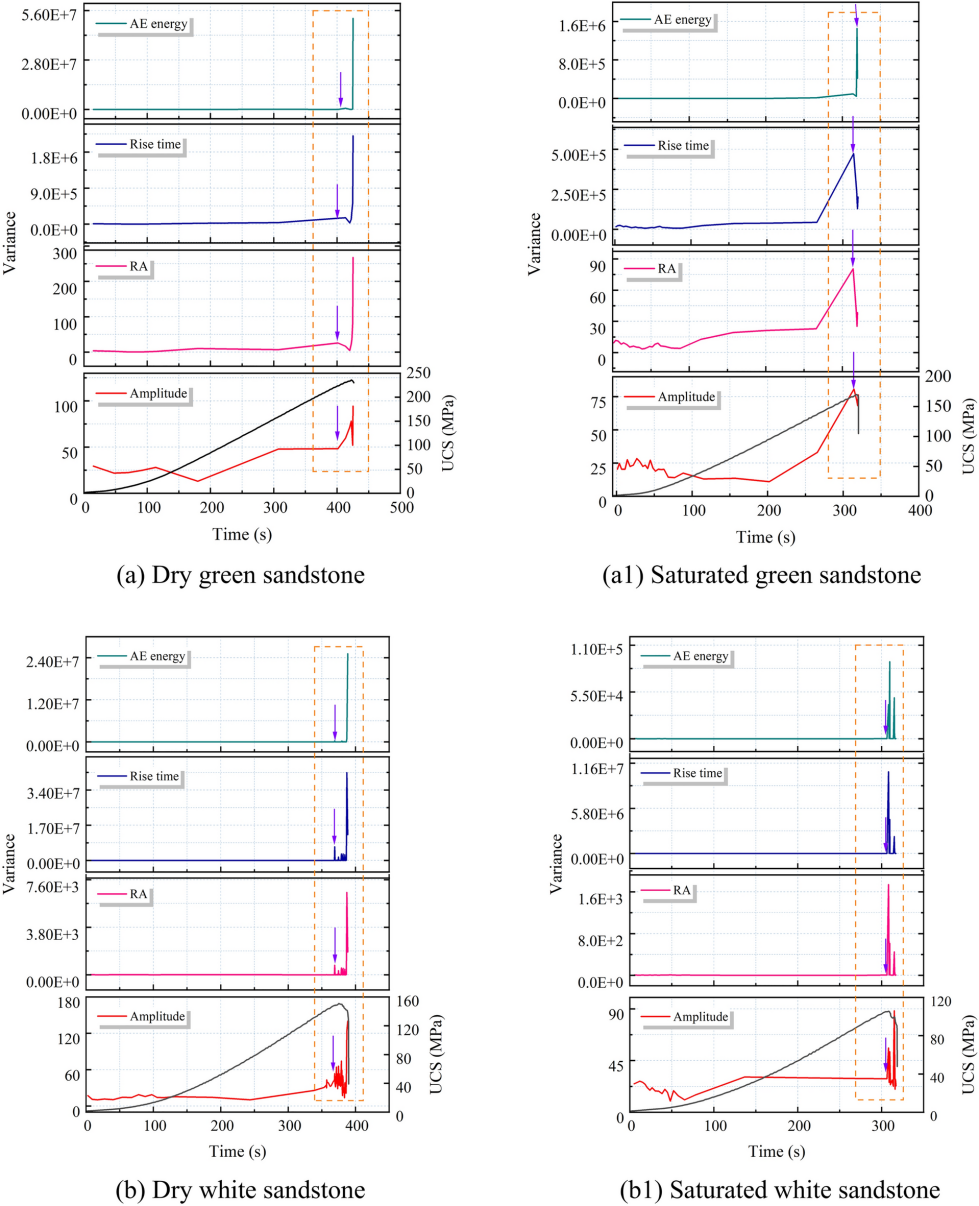

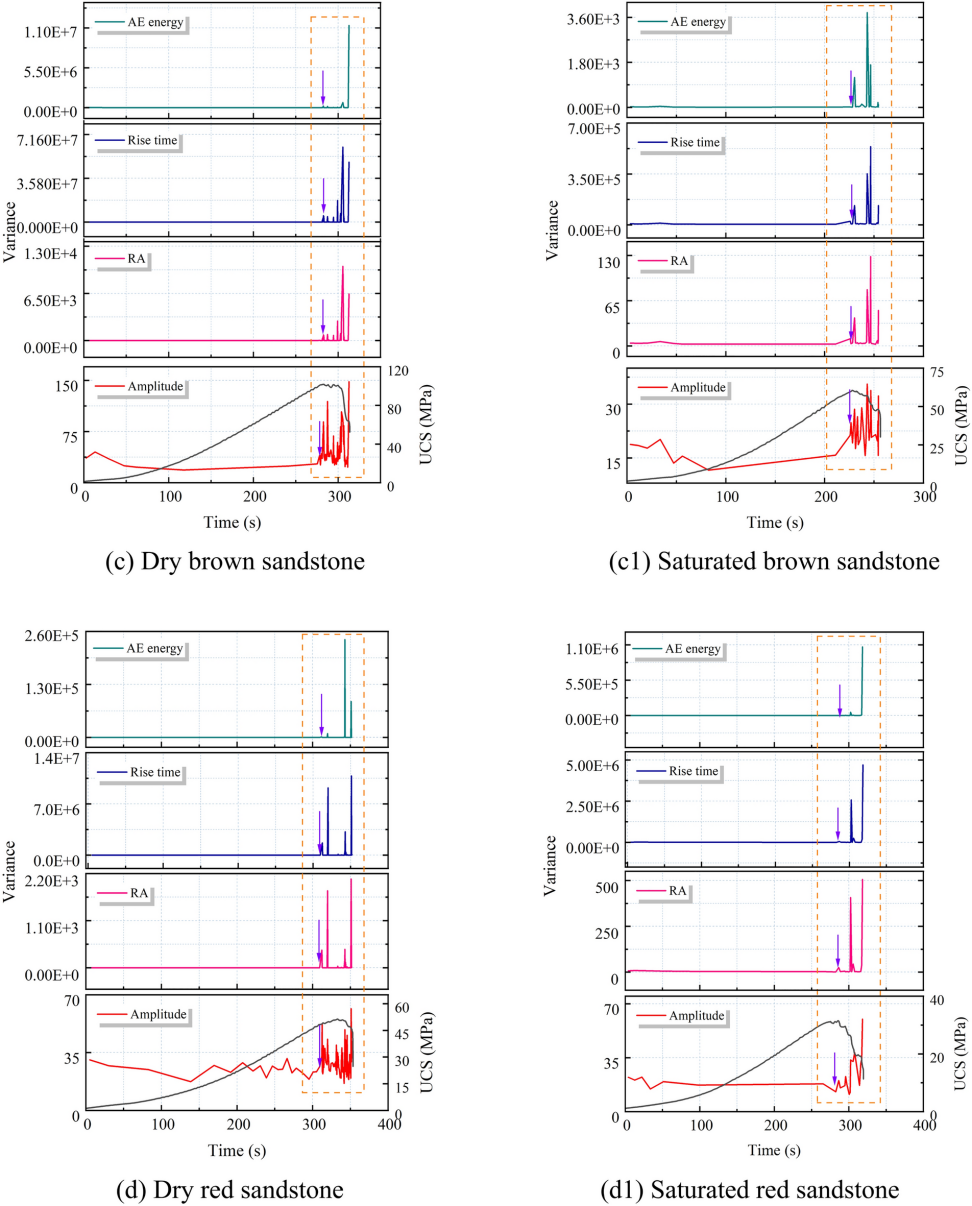
Variance law of AE parameters for dry and saturated sandstone.

According to the critical slowing down theory, the variance trends of four AE parameters were calculated, as depicted in Fig. 13. It is observed that the variance of amplitude, RA value, rise time, and AE energy remains relatively stable during the loading process but sharply increases just before failure. This pattern of change is consistent with that of the autocorrelation coefficients, indicating that the sudden increase in the variance of amplitude, RA value, rise time, and AE energy aligns with the theory of critical slowing down phenomenon. Despite the similar variance trends among the four AE parameters, there are notable differences in their distribution ranges. The variance distribution ranges of amplitude and RA value are between 0 and 10^4^, while those of rise time and AE energy are between 0 and 10^7^. Meanwhile, a significant decrease in variance values is observed under saturated conditions, with the maximum value dropping from 10^7^ to 10^6^. Additionally, the starting point of the variance for AE energy is not clearly defined, posing a challenge for extracting precursor information. Therefore, in terms of the sensitivity and reliability of precursor information regarding rock failure instability, variance of amplitude, RA value, and rise time are superior to that of AE energy.

From Section “Cumulative crack and energy curve characteristics”, it is evident that in the vicinity of macroscopic failure, there is a significant increase in the cumulative number of tensile and shear fractures in sandstone, indicating a substantial change in its fracture mechanism and system phase state. This is reflected in both autocorrelation coefficients and variance analyses of AE parameters. In summary, the autocorrelation coefficients and variance of amplitude, RA value, rise time, and AE energy exhibit rapid and sustained increases in the vicinity of failure, reaching their maximum values at the onset of overall failure, thus serving as precursory features of sandstone macroscopic failure. However, for sandstone with high porosity, the fluctuation of autocorrelation coefficients appears more chaotic compared to variance, resulting in numerous spurious signals. Therefore, variance, being clearer in indicating precursor information compared to autocorrelation coefficients, can be considered the primary criterion, with autocorrelation coefficients serving as supplementary criteria for precursor information.

## Discussion

### The influence of water on sandstone fracture characteristics

**Fig. 14:**
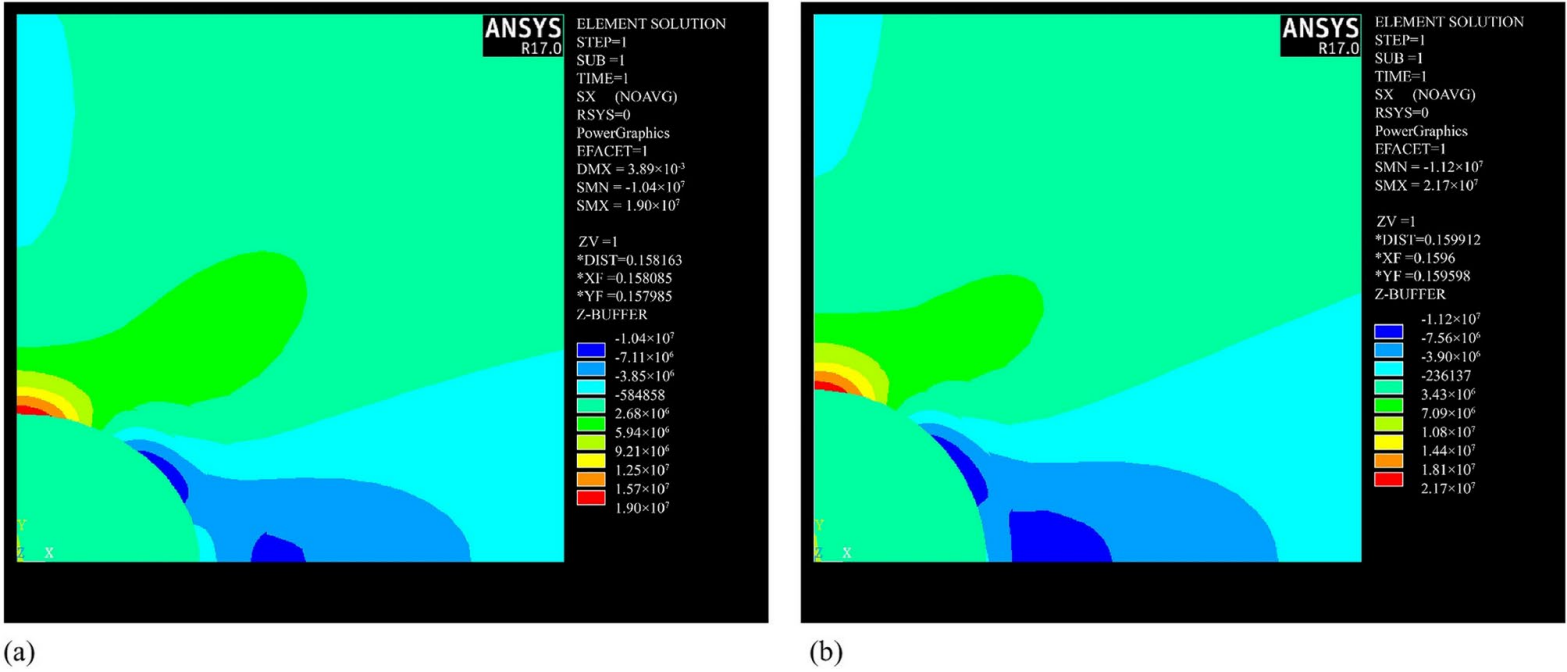
Ansys simulation results of a single void material under axial stress (20 MPa)^34^: (**a**) without pore pressure; (**b**) with pore pressure.

**Fig. 15:**
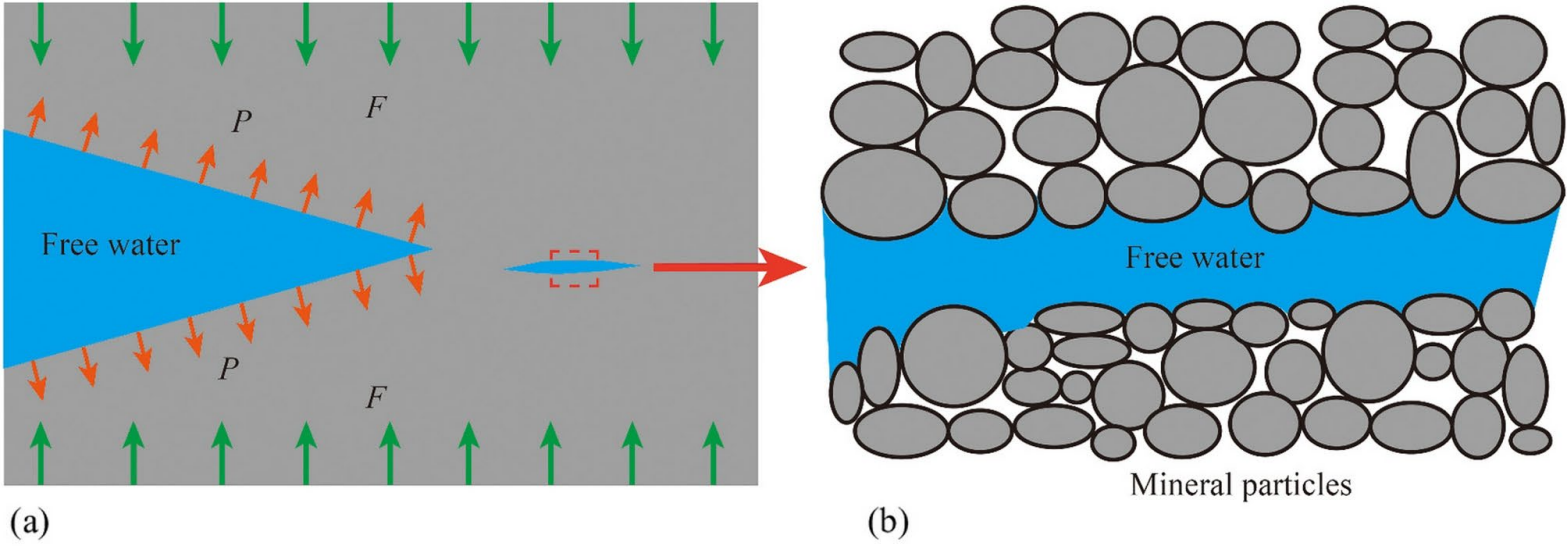
Water–Rock interaction^34^: (**a**) pore water pressure; (**b**) weakening of friction due to lubrication.

The internal mechanism of increased tensile cracking in rocks under saturated conditions can be attributed to the multi-scale synergistic effects induced by water–rock interactions. From a macro-to-micro mechanical behavior analysis, pore water pressure, based on the effective stress principle, reduces the confining pressure on the rock skeleton, leading to a significant increase in the tensile stress around the pores (from 19 MPa in the dry state to 21.7 MPa in the saturated state, a 14.2% increase), causing the stress intensity factor at the crack tip (K_I_) to exceed the critical value (K_IC_), thus driving the preferential initiation of tensile cracks^34^ (Fig. 14). This process is particularly pronounced in red sandstone, which has a higher porosity (15.844%) and better connectivity. The higher pore water pressure transmission efficiency in red sandstone compared to more compact sandstones results in a tensile crack growth rate of 17.53%. Meanwhile, water molecules form adsorption layers at the mineral interfaces, reducing the friction coefficient between particles through physical lubrication and weakening shear resistance (Fig. 15). Clay minerals undergo lattice expansion and cementation disintegration upon water exposure, further compromising the integrity of the microstructure. This coupled mechanism of pore water pressure, mineral softening, and friction weakening promotes a shift in the failure mode from shear-dominated to tensile-dominated. In the initial stage, pore water pressure induces localized tensile stress concentration; in the propagation stage, water infiltration accelerates the crack front’s penetration, ultimately resulting in a failure mode dominated by tensile rupture surfaces.

### Response mechanism of resistivity under uniaxial loading of sandstone

Research shows that during the fracturing process of rock under stress, the initiation and propagation of internal cracks rearrange the pore structure and alter the conductive network, leading to significant changes in resistivity. The results of this experiment indicate that the resistivity of sandstone is primarily influenced by porosity and the water content within the pores. According to Maxwell’s equation, the theoretical formula for the conductivity of a medium composed of two materials can be written as^35^:

9 $${G}_{\text{R}}={G}_{1}\frac{3{G}_{2}-2\Phi ({G}_{2}-{G}_{1})}{3{G}_{1}+\Phi ({G}_{2}-{G}_{1})}$$

where ${G}_{2}$ and ${G}_{1}$ are the conductivities of the two materials, respectively. When these materials combine to form a composite medium, the total conductivity is represented by ${G}_{\text{R}}$. Additionally, $\Phi$ represents the volume fraction of material 1 in the entire medium, defined as $\Phi ={V}_{1}/V$.

In saturated sandstone, materials 1 and 2 are defined as water and ideal sandstone, respectively. ${G}_{\text{w}}$ and ${G}_{\text{s}}$ represent the conductivities of water and sandstone, respectively. Since ${G}_{\text{W}}$ ≫ ${G}_{\text{S}}$, we assume $\frac{{G}_{\text{S}}}{{G}_{\text{W}}}=0$. Thus, Eq. (9) can be written as:

10 $${G}_{\text{Rs}}={G}_{\text{w}}\frac{3{G}_{\text{s}}-2\Phi ({G}_{\text{s}}-{G}_{\text{w}})}{3{G}_{\text{w}}+\Phi ({G}_{\text{s}}-{G}_{\text{w}})}={G}_{\text{w}}\frac{3{G}_{\text{s}}+2\Phi {G}_{\text{w}}}{3{G}_{\text{w}}-\Phi {G}_{\text{w}}}={G}_{\text{w}}\frac{2\Phi }{3-\Phi }$$

where ${G}_{\text{Rs}}$ is the conductivity of the saturated composite medium, and $\Phi$ represents the porosity of the sandstone. It is evident that the resistivity of saturated sandstone is closely linked to its pore structure. When sandstone reaches saturation and enters the plastic deformation stage, the generation and propagation of new cracks cause a water film to rapidly cover the surfaces of pores and cracks. This process increases the conductivity of saturated sandstone, particularly red sandstone, leading to a corresponding decrease in resistivity.

In dry sandstone, materials 1 and 2 are defined as ideal sandstone and air, respectively. ${G}_{\text{A}}$ and ${G}_{\text{s}}$ represent the conductivities of air and sandstone, respectively. Since ${G}_{\text{S}}\gg {G}_{\text{A}}$, we assume $\frac{{G}_{\text{A}}}{{G}_{\text{S}}}=0$. Thus, Eq. (9) can be written as:

11 $${G}_{\text{Rd}}={G}_{\text{A}}\frac{3{G}_{\text{S}}-2\Phi ({G}_{\text{S}}-{G}_{\text{A}})}{3{G}_{\text{A}}+\Phi ({G}_{\text{S}}-{G}_{\text{A}})}={G}_{\text{A}}\frac{3{G}_{\text{s}}-2\Phi {G}_{\text{s}}}{3{G}_{\text{A}}+\Phi {G}_{\text{s}}}={G}_{\text{A}}\frac{3-2\Phi }{\Phi }$$

where ${G}_{\text{Rd}}$ is the conductivity of the dry composite medium, and $\Phi$ represents the porosity of the sandstone. It can be observed that in the plastic deformation stage, the expansion of fractures in sandstone increases porosity, leading to a decrease in conductivity and an increase in resistivity. This phenomenon is consistent with the trend observed in uniaxial compression tests, where resistivity increases with the unstable expansion of fractures.

Overall, the changes in resistivity during the fracturing process of sandstone are closely related to its pore structure. Under low pressure, the primary cracks in the sandstone close, reducing porosity and changing resistivity. In the high-stress stage, various degrees of fractures and rupture surfaces develop within the sandstone. The presence of air or the rapid filling of these new fractures with water directly alters the conduction mechanism, resulting in different resistivity trends.

### The Precursory information characteristics of sandstone’s critical slowing down

**Fig. 16:**
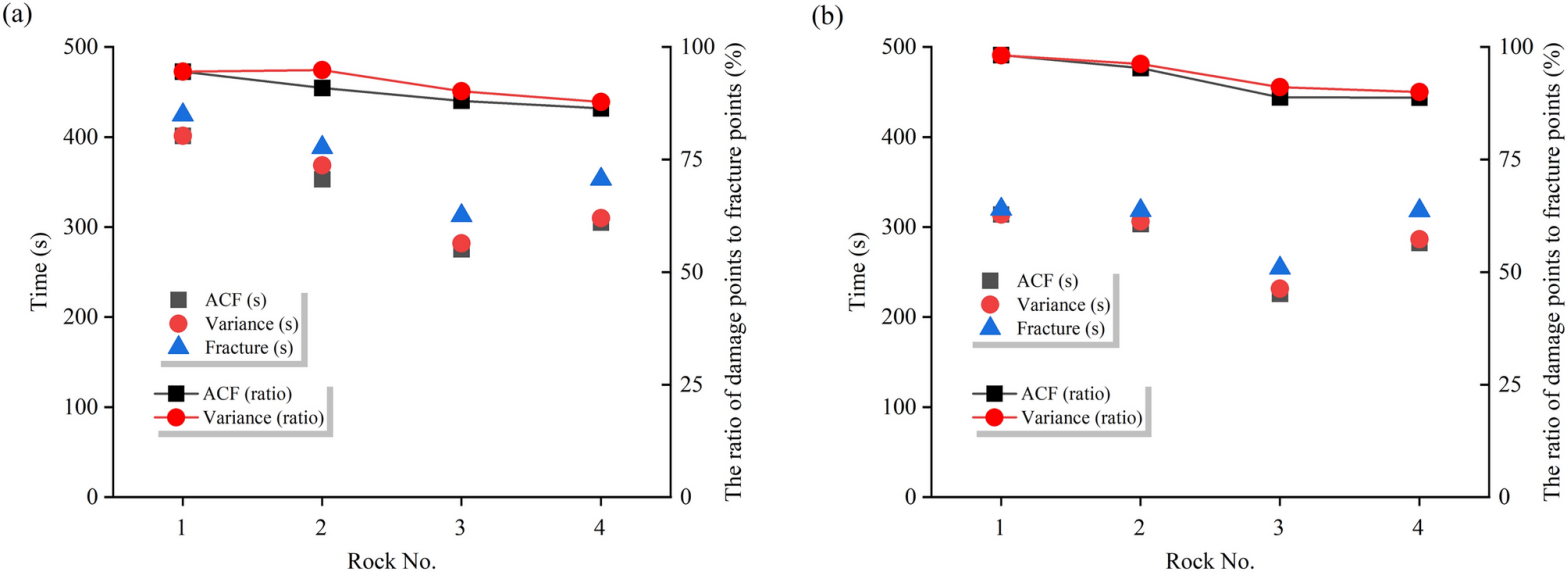
Precursor information of critical slowing down in sandstone: (**a**) dry; (**b**) saturated.

The application of critical slowing down theory in the field of rock engineering prediction provides a new method for predicting rock failure based on AE signal analysis. As demonstrated in section “Critical slowing down characteristics of sandstones” of this paper, during the loading process of sandstone, critical slowing down phenomena are observed in the signals of amplitude, RA value, rise time, and AE energy. Specifically, both the autocorrelation function (ACF) and variance (V) exhibit a sharp increase before the instability failure of the rock sample, serving as precursor signals of impending failure. This theoretical framework finds support in the research on rock failure prediction. In order to identify and compare the effectiveness of these indicators in predicting the failure of saturated sandstone, a comprehensive analysis of these parameters is conducted in this section. The time points where ACF and V exhibit significant changes are considered as “critical damage precursor points” and denoted as D, while the time of complete failure of the sample is denoted as F. Their ratio, denoted as W, serves as a damage warning indicator, used to analyze the timeliness of sandstone fracture prediction. Since the ACF and V of the four AE parameters demonstrate equal sensitivity and reliability, the rise time is chosen as the representative parameter. Table 3 and Fig. 16 present the precursor information for different sandstone failures.

**Table 3:** Precursor warning information of critical slowing down theory and correlation dimension.

Rock No	Rocks	Water content state	Critical damage precursor points (s)		Complete failure (s)	Ratio (Damage warning indicator)/%	
			ACF	V		ACF	V
1	Green sandstone	Dry	401.28	401.22	424.55	94.52	94.50
		Saturated	313.98	313.96	319.81	98.18	98.17
2	White sandstone	Dry	353.02	368.55	388.43	90.88	94.88
		Saturated	303.36	306.36	318.35	95.29	96.23
3	Brown sandstone	Dry	275.31	281.99	312.71	88.04	90.18
		Saturated	225.86	231.65	254.37	88.79	91.07
4	Red sandstone	Dry	304.98	309.98	353.14	86.36	87.78
		Saturated	282.4	286.36	318.21	88.75	89.99

Table 3 reveals that for dry green sandstone, both ACF and V exhibit the same W value, standing at 94.5%. Saturated green sandstone demonstrates W values of 98.18%, 98.17%, and 97.2% respectively. It is noteworthy that while ACF and V share identical W values, the W values under saturation increase by 3.8%, indicating a shifting of the transition point time of autocorrelation and variance towards complete failure. This pattern is consistent among other water-containing sandstones. The rationale lies in the fact that water amplifies the attenuation of AE signals, as water molecules absorb acoustic energy to a greater extent than solid particles in dry rocks. Consequently, under saturation, acoustic signals require more time to attenuate below the threshold triggering an alarm, leading to a posterior shift in the transition point time. Moreover, the W values of green sandstone, white sandstone, brown sandstone, and red sandstone are inversely correlated with porosity. From the perspective of microscopic micro-fracturing, higher porosity implies a greater abundance of minute cracks and pores within the internal microstructure of sandstone. When subjected to external stress, these tiny cracks and pores serve as nucleation points for micro-crack initiation, thereby promoting the propagation of micro-cracks in sandstone, ultimately causing an earlier transition in the system’s phase. Simultaneously, sandstones with higher porosity manifest multiple false signals in autocorrelation coefficients before imminent failure, exacerbating the difficulty in extracting precursor information (Section “Autocorrelation coefficient characteristics of AE parameters”).

In summary, it can be concluded that amplitude, RA value, rise time, and AE energy all exhibit a critical slowdown phenomenon characterized by an increase in variance and autocorrelation coefficients as failure approaches. These serve as precursor signals to sandstone failure, albeit influenced by water content and porosity.

## Conclusion

This study conducted uniaxial compression tests on four types of saturated sandstones (green sandstone, white sandstone, brown sandstone, and red sandstone). Acoustic Emission (AE) and resistivity measurements were simultaneously observed throughout the loading process. The fracturing behavior of the saturated sandstones was analyzed using AE energy, resistivity ratio, and RA–AF. The critical slowdown theory was employed to analyze the autocorrelation coefficient and variance features of acoustic emission parameters such as RA, rise time, amplitude, and energy. The following conclusions were drawn:

1. Under saturated conditions, the uniaxial compressive strength and elastic modulus of different types of sandstone (green sandstone, white sandstone, brown sandstone, red sandstone) both exhibit significant decreases. The softening coefficients are 0.71, 0.68, 0.59, 0.65; 0.91, 0.83, 0.8, 0.81, respectively. The changes in sandstone resistivity sensitively reflect variations in internal porosity under loading, as well as the closure of microcracks and the initiation and propagation of fractures. Furthermore, the variations in resistivity exhibit good complementarity with the characteristics of acoustic emission energy.
2. The accumulated acoustic emission energy values of saturated sandstone are significantly lower than those in the dry state, indicating that the presence of water diminishes the cumulative damage within the sandstone. The crack classification results of RA–AF are consistent with the macroscopic failure morphology. Meanwhile, the tensile cracks in the water-containing sandstone grew by 13.66%, 13.69%, 14.86%, and 17.53%, respectively. This means that the presence of water promotes the formation of tensile cracks, and the expansion of cracks is closely related to the porosity of sandstone, primarily controlled by pore water pressure.
3. The autocorrelation coefficients and variances of acoustic emission parameters (amplitude, RA value, rise time, energy) sharply increase before critical failure, exhibiting critical slowdown phenomena, serving as a precursor signal of rock failure. Among these, variance, compared to autocorrelation coefficients, provides clearer precursor information, thus serving as the primary criterion for precursor information, while autocorrelation coefficients serve as auxiliary criteria.
4. The presence of water increases the attenuation of acoustic emission signals, resulting in the postponement of damage precursor indicators under saturated conditions. Additionally, sandstone with higher porosity generates multiple false signals before approaching failure, increasing the difficulty of extracting precursor information. Therefore, the sensitivity and reliability of critical slowing down theory warning are influenced by water and porosity and should be considered in practical applications.

## Data Availability

Some or all data, or code generated or used during the study are available from the corresponding author by request (i.e., raw results).
